# COVID-19 vaccine acceptance among pregnant women and mothers of young children: results of a survey in 16 countries

**DOI:** 10.1007/s10654-021-00728-6

**Published:** 2021-03-01

**Authors:** Malia Skjefte, Michelle Ngirbabul, Oluwasefunmi Akeju, Daniel Escudero, Sonia Hernandez-Diaz, Diego F. Wyszynski, Julia W. Wu

**Affiliations:** 1 Department of Global Health and Population, Harvard TH Chan School of Public Health, Boston, MA USA; 2 Department of Epidemiology, Harvard TH Chan School of Public Health, Boston, MA USA; 3 Pregistry, Los Angeles, CA USA

**Keywords:** Vaccine acceptance, Vaccine confidence, COVID-19, Pregnancy, Vaccination

## Abstract

With the development of multiple effective vaccines, reducing the global morbidity and mortality of COVID-19 will depend on the distribution and acceptance of COVID-19 vaccination. Estimates of global vaccine acceptance among pregnant women and mothers of young children are yet unknown. An understanding of the challenges and correlates to vaccine acceptance will aid the acceleration of vaccine administration within these populations. Acceptance of COVID-19 vaccination among pregnant women and mothers of children younger than 18-years-old, as well as potential predictors, were assessed through an online survey, administered by Pregistry between October 28 and November 18, 2020. 17,871 total survey responses from 16 countries were obtained. Given a 90% COVID-19 vaccine efficacy, 52.0% of pregnant women (n = 2747/5282) and 73.4% of non-pregnant women (n = 9214/12,562) indicated an intention to receive the vaccine. 69.2% of women (n = 11,800/17,054), both pregnant and non-pregnant, indicated an intention to vaccinate their children. Vaccine acceptance was generally highest in India, the Philippines, and all sampled countries in Latin America; it was lowest in Russia, the United States and Australia. The strongest predictors of vaccine acceptance included confidence in vaccine safety or effectiveness, worrying about COVID-19, belief in the importance of vaccines to their own country, compliance to mask guidelines, trust of public health agencies/health science, as well as attitudes towards routine vaccines. COVID-19 vaccine acceptance and its predictors among women vary globally. Vaccination campaigns for women and children should be specific for each country in order to attain the largest impact.

**Supplementary Information:**

The online version contains supplementary material available at 10.1007/s10654-021-00728-6.

## Background

As we reach the one-year anniversary of the Severe Acute Respiratory Syndrome Coronavirus 2 (SARS-CoV-2) pandemic, the devastating impacts of this infectious disease outbreak on society are clear. By the end of 2020, the total number of Coronavirus Disease 2019 (COVID-19) cases reached more than 80 million worldwide, and the number of deaths from COVID-19 related causes hit 1.8 million [1]. Along with the loss of human life, the pandemic has led to unprecedented public health challenges, stressing health care systems, disrupting supply chains and economies, and prompting a mental health crisis [2, 3]. Simultaneously, several COVID-19 vaccines have been developed and approved at an unprecedented speed, while holding rigorous regulatory processes [4–6]. These vaccines, however, cannot curb the epidemic without widespread acceptance. Assuming a basic reproductive number of 4, the community immunity level needs to reach at least 75% to stop the COVID-19 pandemic. Therefore, we must consider vaccine delivery strategies and determine the vaccine acceptance needed for society to return to pre-pandemic conditions [7]. The World Health Organization (WHO) has listed vaccine hesitancy, defined as the delay in acceptance or refusal of vaccines, as one of the top ten threats to global health, even prior to the current COVID-19 pandemic [8, 9]. Early COVID-19 vaccine surveys on vaccine acceptance foreshadow global challenges to COVID-19 vaccine distribution [10–12].

The COVID-19 vaccine trials generated very limited data on safety and efficacy for pregnant women and children. Yet, pregnant women with symptomatic COVID-19 might be at an increased risk for severe illness than non-pregnant peers; and children appear to have a lower risk for symptomatic COVID-19 but similar rates of infection, making them potential transmitters of the virus [13–19]. Since mothers are often key decision-makers for whether their children will receive vaccinations, it is important to measure vaccine confidence among mothers of young children, and to investigate the predictors for their vaccine acceptance or reluctance, in order to prepare for COVID-19 vaccination efforts. In this study, we describe the level of acceptance, attitudes surrounding the COVID-19 vaccine and key predictors of COVID-19 vaccine acceptance among pregnant women and mothers of young children in 16 countries.

## Methods

### Study design and data collection

**Fig. 1:**
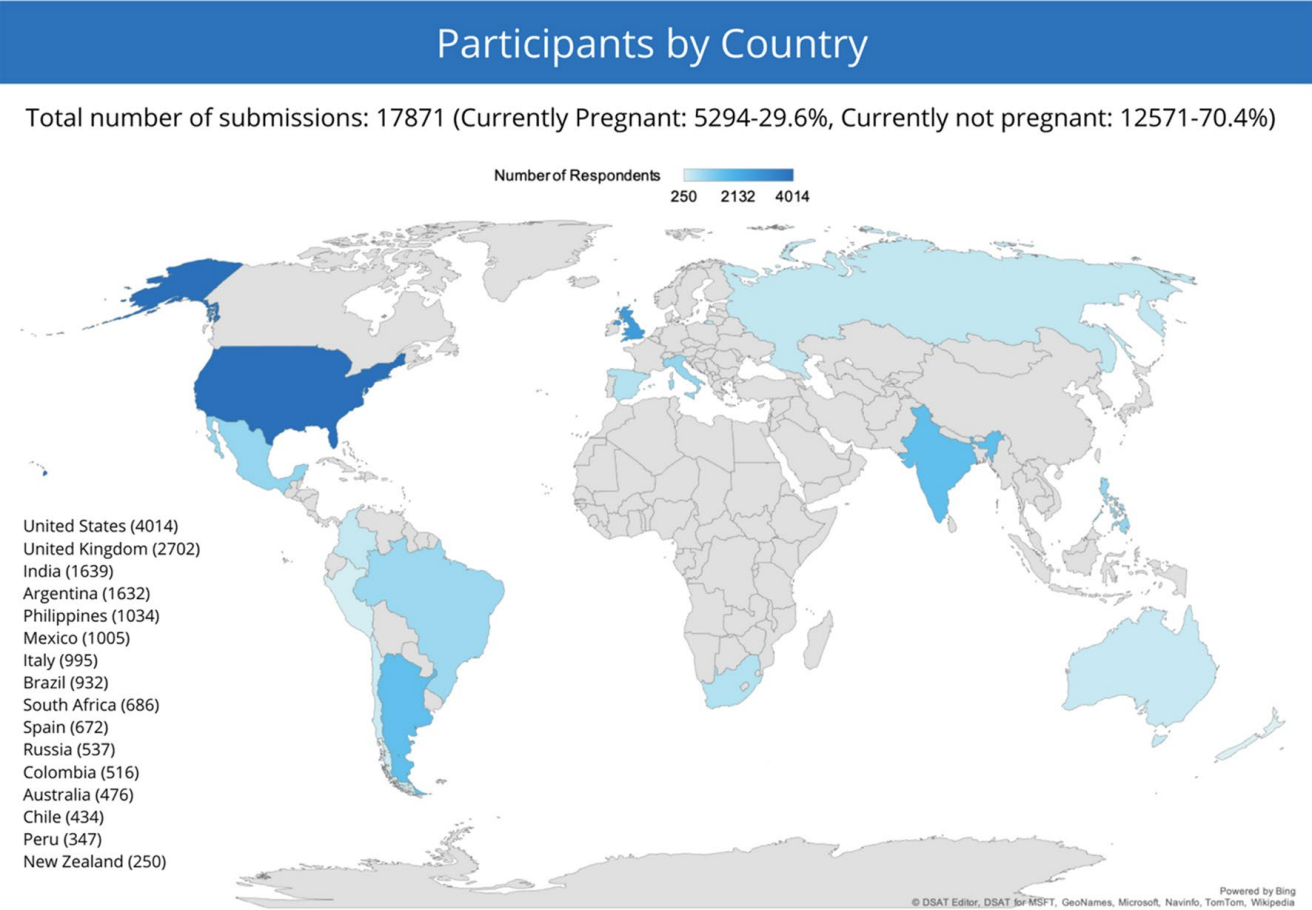
Enrollment occurred in the 14 most-affected countries during Oct 28–Nov 18, 2020, through an online pregnant women and mothers’ social media community, Pregistry. New Zealand and Australia were included as reference countries

An anonymous, online, cross-sectional survey was conducted in 16 countries between October 28 and November 18, 2020, to assess the COVID-19 vaccine acceptance level and potential predictors among pregnant women and mothers of young children for themselves and for their children. Predictors examined included COVID-19 vaccine confidence, negative experiences with COVID-19, perception on the risk and seriousness of COVID-19, public trust, general vaccine attitude, demographics and socioeconomic status. This survey included women aged 18 years or older, currently pregnant or with at least one child under 18 years of age. All potential participants were informed of the research objectives as well as the standards of data confidentiality. Fourteen countries with high cumulative incidences of COVID-19 as of Oct 28, 2020 were included, with their ranking: the United States (US), India, Brazil, Russia, Spain, Argentina, Colombia, UK, Mexico, Peru, South Africa, Italy, Chile and the Philippines. Additionally, Australia and New Zealand were included for reference given that they had lower incidences of COVID-19 (Fig. 1). The survey was hosted on the Pregistry platform for COVID-19 studies and advertised predominantly on social media platforms and online parenting forums [20]. The advertisements and survey were available in six languages (English, French, Italian, Portuguese, Russian, and Spanish). Interested participants were invited to follow a link to take the survey. Additional details on the survey structure can be found in the supplemental materials.

### Definition of COVID-19 vaccine acceptance

To measure vaccine acceptance among three groups (pregnant women, non-pregnant women and children), three separate questions were used. Each question followed a general format “If a COVID-19 vaccine were safe and available to (you/your child/children) for free, how likely would (you/your child/children) be to get vaccinated (during pregnancy) if the vaccine has an efficacy of [90% (in other words, it reduces the chance of getting infected by 90%)?]. The first two questions regarding self-vaccination could only be answered by pregnant women and non-pregnant women, respectively. The third question regarding child-vaccination was open to all women. For each question, responses were recorded on a five-point Likert scale (‘very likely’, ‘fairly likely’, ‘somewhat likely’, ‘quite unlikely’ and ‘not at all likely’). Vaccine acceptance was defined as yes if a respondent answered, ‘very likely’, ‘fairly likely’ or ‘somewhat likely’, and no for any other responses.

### Statistical analysis

We tabulated the distribution of the COVID-19 vaccine acceptance responses against questions from the predictor sections. Denominators vary slightly due to missingness in participant responses. We ran univariate logistic regressions to identify potential predictors of women’s COVID-19 vaccine acceptance/reluctance across countries. Strongest predictors as well as key demographic factors were included in the multivariate regression models. The area under the receiver operating characteristic (ROC) curve (AUC) was used to assess models’ prediction capacity. We further examined differences in attitudes and acceptance between countries and repeated the prediction models within countries.

## Findings

### Study participants

Among 17,871 respondents, 29.6% (n = 5294) were pregnant at the time of taking the survey. Among pregnant women, 49.7% (n = 2629) had at least one child (Table 1). For those who were pregnant, the mean gestational age was 20.0 weeks (SD = 9.4). The mean age of all respondents, both pregnant and non-pregnant, was 34.4 years (SD = 7.3). A summary of respondent demographics is included in Table 1. Country-specific demographics are summarized in Table 1S. Overall distributions of age were homogenous across countries. India, Italy, Australia and New Zealand had higher than overall proportions of high-income respondents; US, South Africa, UK, India, Australia and New Zealand had higher than overall proportions of highly educated respondents; US, Russia and India had higher than overall proportions of married respondents.

**Table 1:** Baseline characteristics among pregnant women and mothers of young children (n = 17,871)

Vaccine acceptance among core demographic groups	All respondents N = 17,871 (100)	Accept for themselves				Accept for child/children	
		Pregnant women N = 5282		Non-pregnant mothers N = 12,562		Women who indicated whether or not they would vaccinate their child/children N = 17,054	
	Total frequency (% of sample)	All N (%)	Accepted vaccine N (%)	All N (%)	Accepted vaccine N (%)	All N (%)	Accepted vaccine N (%)
No. children							
None	2661 (14.9)	2658 (50.3)	1488 (56.0)	NA	NA	1916 (11.2)	1416 (73.9)
One child	6795 (38.1)	1735 (32.9)	906 (52.2)	5054 (40.2)	3957 (78.3)	6765 (39.7)	4878 (72.1)
Two children	5465 (30.6)	594 (11.3)	249 (41.9)	4868 (38.8)	3599 (73.9)	5448 (32.0)	3785 (69.5)
At least 3 children	2938 (16.5)	295 (5.6)	104 (35.3)	2640 (21.0)	1658 (62.8)	2925 (17.2)	1721 (58.8)
Age (years)							
18–24	1299 (7.3)	674 (12.8)	331 (49.1)	625 (5.0)	415 (66.4)	1175 (6.9)	716 (60.9)
25–29	3396 (19.0)	1566 (29.7)	807 (51.5)	1827 (14.5)	1287 (70.4)	3118 (18.3)	2050 (65.8)
30–34	4937 (27.6)	1911 (36.2)	1010 (52.9)	3021 (24.1)	2235 (74.0)	4680 (27.4)	3254 (69.6)
35–39	4041 (22.6)	983 (18.6)	518 (52.7)	3054 (24.3)	2247 (73.6)	3929 (23.0)	2749 (70.0)
40–65	4189 (23.5)	148 (2.8)	81 (54.7)	4035 (32.1)	3030 (75.1)	4152 (24.4)	3031 (73.0)
Socioeconomic status							
Poor	1993 (11.2)	444 (8.4)	249 (56.1)	1548 (12.3)	1054 (68.1)	1944 (11.4)	1264 (65.0)
Lower-middle class	5224 (29.3)	1386 (26.2)	727 (52.5)	3835 (30.5)	2757 (71.9)	5027 (29.5)	3446 (68.6)
Middle class	8061 (45.2)	2553 (48.3)	1295 (50.7)	5505 (43.8)	4107 (74.6)	7662 (44.9)	5345 (69.8)
Upper-middle class or wealthy	2573 (14.4)	899 (17.0)	476 (53.0)	1674 (13.3)	1296 (77.4)	2421 (14.2)	1745 (72.1)
Education							
Never attended high school	1098 (6.2)	239 (4.5)	104 (43.5)	859 (6.9)	614 (71.5)	1074 (6.3)	698 (65.0)
High school or GED	2114 (11.9)	469 (8.9)	263 (56.1)	1644 (13.1)	1151 (70.0)	2061 (12.1)	1382 (67.1)
Some College or University	3159 (17.7)	867 (16.4)	422 (48.7)	2289 (18.3)	1575 (68.8)	3052 (17.9)	1964 (64.4)
College Diploma or University Degree	6746 (37.9)	2164 (41.0)	1092 (50.5)	4579 (36.5)	3304 (72.2)	6386 (37.5)	4337 (67.9)
Master's, Professional or Doctoral Degree	4703 (26.4)	1534 (29.1)	862 (56.2)	3167 (25.3)	2558 (80.8)	4450 (26.1)	3403 (76.5)
Health insurance status							
Insured	13,737 (77.0)	4416 (83.6)	2284 (51.7)	9319 (74.2)	6888 (73.9)	13,050 (76.5)	9090 (69.7)
Not insured	4111 (23.0)	866 (16.4)	263 (53.5)	3243 (25.8)	2326 (71.7)	4004 (23.5)	2710 (67.7)
Marital status							
Married	12,004 (67.4)	3762 (71.4)	1934 (51.4)	8236 (65.8)	6094 (74.0)	11,456 (67.4)	7955 (69.4)
All unmarried	5797 (32.6)	1505 (28.6)	806 (53.6)	4288 (32.2)	3095 (72.1)	5546 (32.6)	3816 (68.8)
Living with a partner	3195 (18.0)	1031 (19.6)	580 (56.3)	2162 (17.3)	1623 (75.1)	3026 (17.8)	2163 (71.5)
Never married	1541 (8.7)	386 (7.3)	188 (48.7)	1154 (9.2)	782 (67.8)	1471 (8.7)	952 (64.7)
Divorced	514 (2.9)	55 (1.0)	26 (47.3)	458 (3.7)	312 (68.1)	507 (3.0)	323 (63.7)
Separated	437 (2.5)	27 (0.5)	12 (44.4)	410 (3.3)	296 (72.2)	342 (2.5)	298 (69.0)
Widowed	110 (0.6)	6 (0.1)	0 (0)	104 (0.8)	82 (78.9)	110 (0.7)	80 (72.7)

### Global vaccine acceptance and confidence

**Fig. 2:**
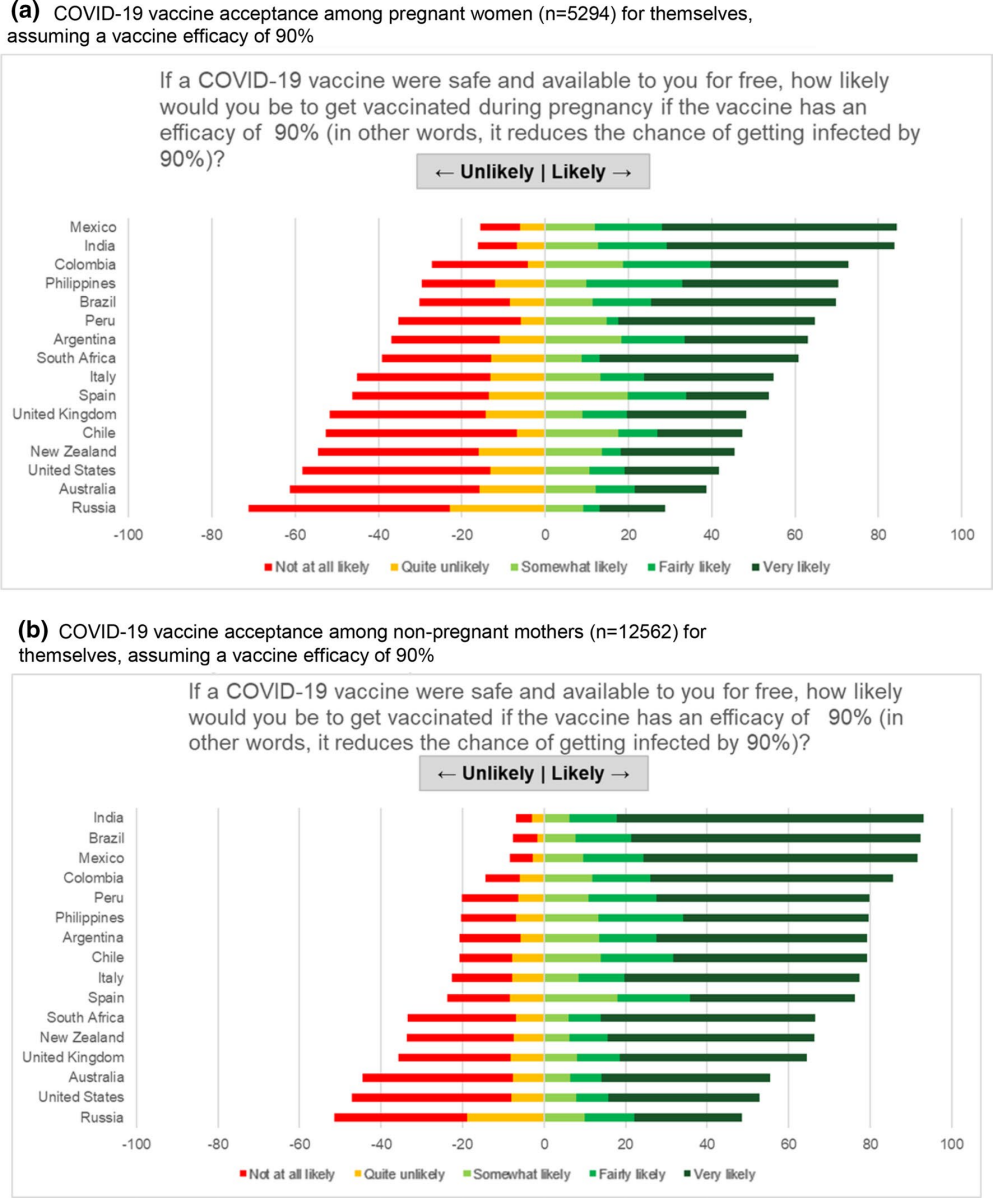

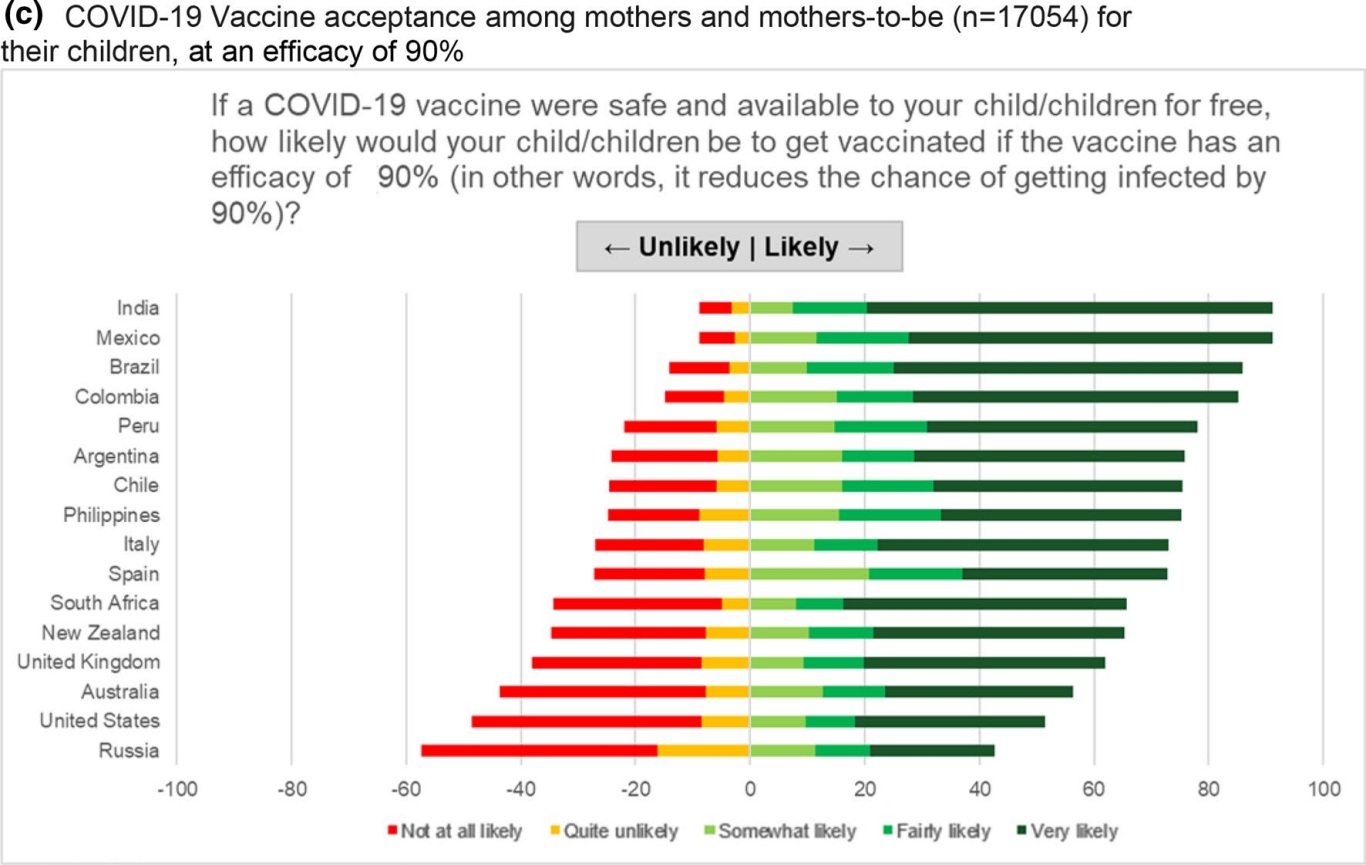
COVID-19 Vaccine acceptance among pregnant women and mothers of young children. **a** COVID-19 vaccine acceptance among pregnant women (n = 5294) for themselves, assuming a vaccine efficacy of 90%. **b** COVID-19 vaccine acceptance among non-pregnant mothers (n = 12,562) for themselves, assuming a vaccine efficacy of 90%. **c** COVID-19 Vaccine acceptance among mothers and mothers-to-be (n = 17,054) for their children, at an efficacy of 90%

Among pregnant women, 52.0% (n = 2747) intended to receive COVID-19 vaccination during their pregnancy if an efficacy of 90% were achieved. Responses among pregnant women varied substantially by country (range: 28.8–84.4%). COVID-19 vaccine acceptance level was above 80% for pregnant women in Mexico and India; and below 45% for US, Australia and Russia (Fig. 2a). Among non-pregnant women, 73.4% (n = 9214/12,562) intended to receive vaccination. COVID-19 vaccine acceptance among non-pregnant women also varied substantially between countries (range 48.6–93.1%). COVID-19 vaccine acceptance level was above 90% for non-pregnant mothers in India, Brazil and Mexico; and below 56% for Australia, US and Russia (Fig. 2b). Among the 17,054 women who stated their likelihood to vaccinate their children, results were very similar. COVID-19 vaccine acceptance levels among mothers for their children was above 85% in India, Mexico, Brazil and Colombia; and below 52% for Australia, US and Russia (Fig. 2c). This country-variable pattern persisted after standardizing for key demographics including age, education, income and marital status.

**Fig. 3:**
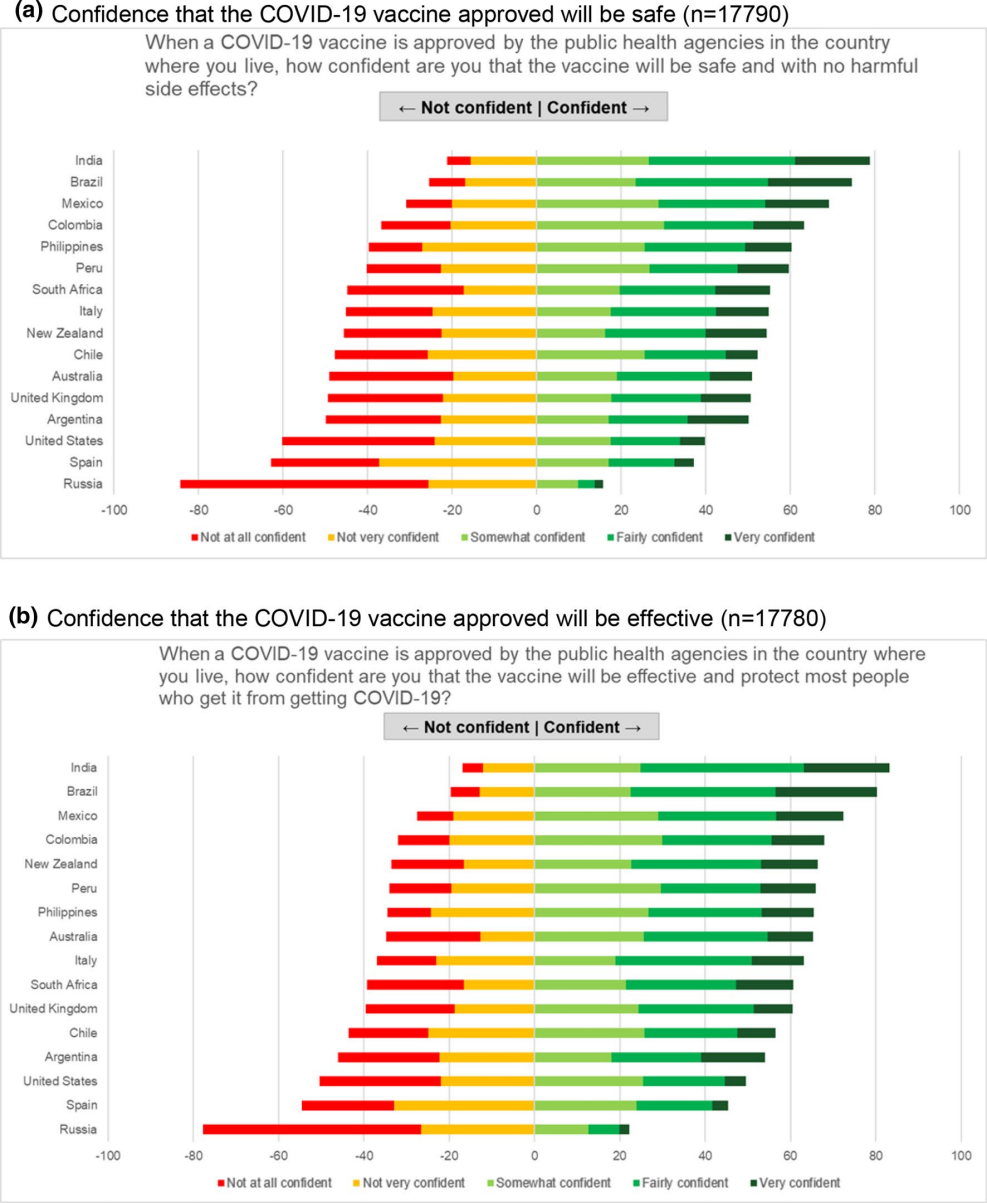
COVID-19 vaccine confidence markers among pregnant women and mothers of young children. **a** Confidence that the COVID-19 vaccine approved will be safe (n = 17,790). **b** Confidence that the COVID-19 vaccine approved will be effective (n = 17,780)

Overall, 53.0% of women were confident that a nationally approved COVID-19 vaccine would be safe, with no harmful side effects (Fig. 3a), and 60.4% were confident that such a vaccine would be effective, protecting most people who receive the vaccine (Fig. 3b).

### Perceptions on the COVID-19 pandemic

**Fig. 4:**
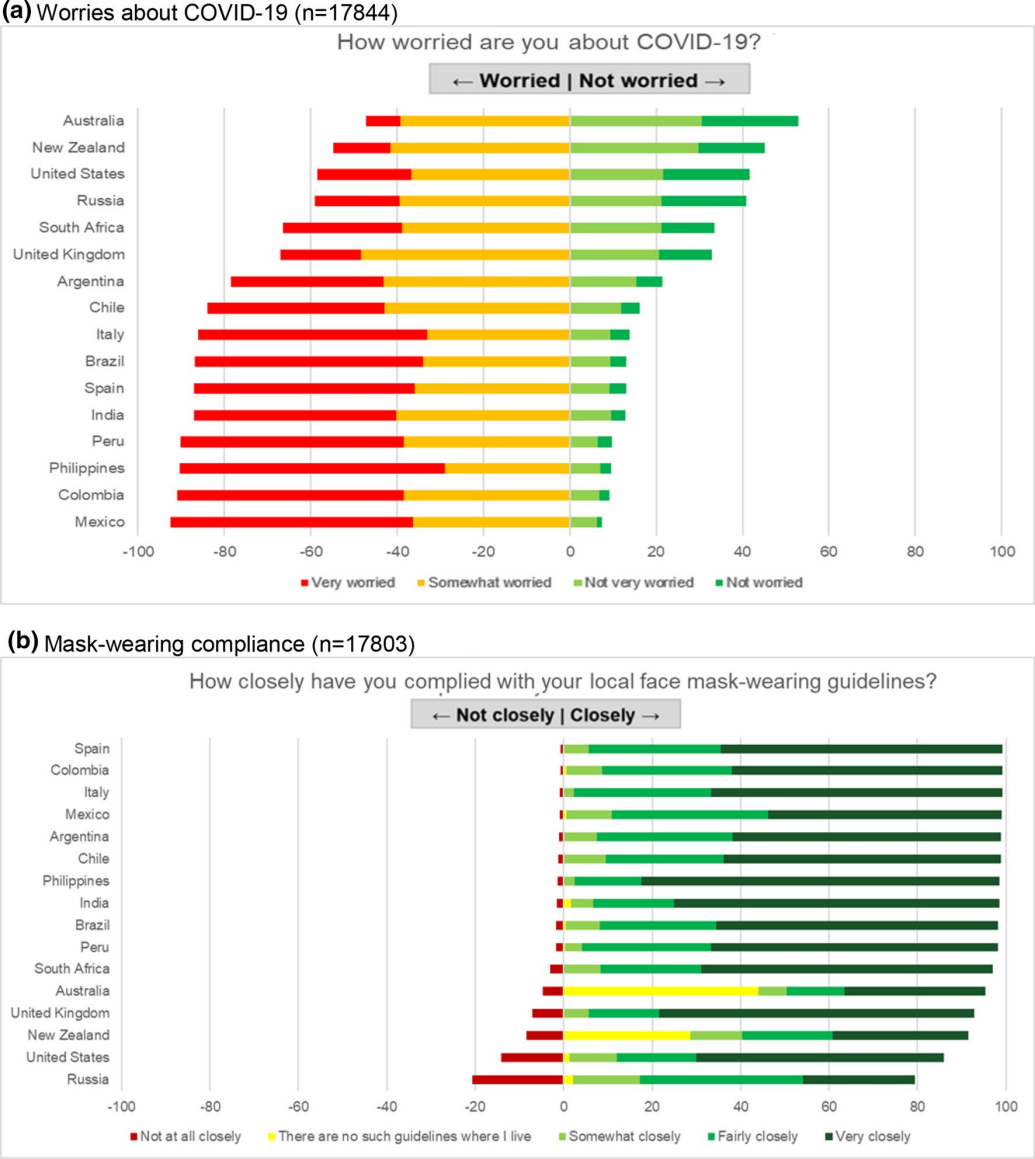
Perceptions of COVID-19 among pregnant women and mothers of school-aged children. **a** Worries about COVID-19 (n = 17,844). **b** Mask-wearing compliance (n = 17,803)

The perceived seriousness of COVID-19 and importance of prevention measures were also highly variable among the 16 sampled countries, and it did not correspond to the infection rate in the country. Women’s level of worry about COVID-19 in the US and Russia was comparable to that in lower incidence countries (Australia and New Zealand) (Fig. 4a). Despite this variation in concern, self-reported compliance with local mask-wearing regulations was above 75% in all countries (Fig. 4b). Most responders trusted health science in general (Figure S2a) and were satisfied with public health authorities in their countries for their performance in controlling the pandemic (Figure S3a); although the trust and satisfaction level varied among countries (Figures S2b, S3b, 5a, b). Though 74.2% of women felt informed on the development of a COVID-19 vaccine (Figure S4), 27.7% did not follow COVID-19 news in any form (TV, radio, newspaper, news websites, social media).

**Fig. 5:**
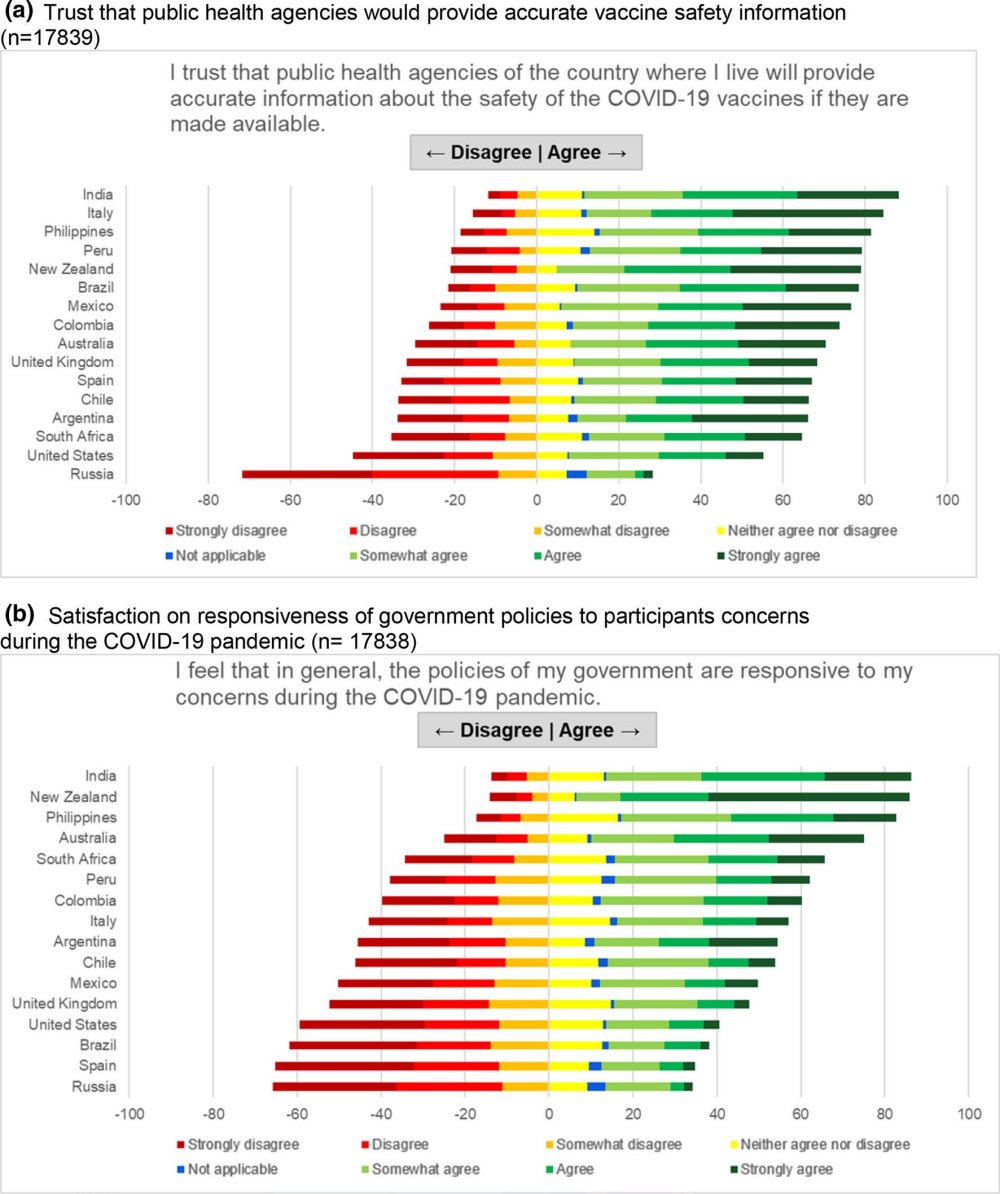
Public trust and satisfaction among pregnant women and mothers of school-aged children. **a** Trust that public health agencies would provide accurate vaccine safety information (n = 17,839). **b** Satisfaction on responsiveness of government policies to participants concerns during the COVID-19 pandemic (n = 17,838)

### Attitudes towards vaccines

The majority of women in the 16 countries believed it was important for their own country to have a COVID-19 vaccine (85.8%) (Figure S1a), and for most people in their own country to get vaccinated (82.6%) (Figure S1b). Perceptions on the importance of childhood vaccinations were also positive, with 92.0% of women responding that vaccines can protect children from serious infectious diseases (Figure S5b). In addition, 49.4%, reported vaccination for influenza in the past year.

### Top reasons for COVID-19 vaccine reluctance

The top three reasons for pregnant women to decline COVID-19 vaccination during pregnancy even if the vaccine were safe and free were that they did not want to expose their developing baby to any possible harmful side effects (65.9%), were concerned that approval of the vaccine would be rushed for political reasons (44.9%) and would like to see more safety and effectiveness data among pregnant women (48.8%). The top reasons for mothers to be unwilling to have their child/children vaccinated for COVID-19 were that they are concerned that approval of the vaccine will be rushed for political reasons (39.8%), would like to see more safety and effectiveness data among children (32.7%), and believe that the vaccine is not safe and could have harmful side effects (28.4%). Health care providers had a limited impact: only 45.9% of pregnant women and 54.6% of non-pregnant women would be more likely to have themselves/children vaccinated if recommended by health care providers.

Overall women indicated higher likelihoods to get vaccinated with higher vaccine efficacies. A sensitivity analysis was conducted to see if there was any difference in vaccine acceptance within-country before and after November 9^th^, 2020, the day in which Pfizer-BioNTech announced news of the first COVID-19 vaccine efficacy results. No significant difference was found in vaccine acceptance outcomes from this test.

### Predictors of COVID-19 vaccine acceptance

Crude-level associations between women’s COVID-19 vaccine acceptance and potential predictors are shown in Table 2. Demographic factors, such as younger age, lower income, lower education level, non-married and no health insurance were slightly linked to COVID-19 vaccine non-acceptance. Strongest predictors of COVID-19 vaccine acceptance were confidence in COVID-19 vaccine safety and efficacy, belief in the importance of vaccines/mass vaccination to their own country, confidence in routine childhood vaccines, worried about COVID-19, trust of public health agencies/health science, as well as compliance to mask guidelines (Figure S6). Although some of these determinants were correlated and their odds ratios diminished in multivariate regression models, they remained the strongest predictors (Table 3). These predictors were similar for pregnant and non-pregnant women, for self-vaccination and for child vaccination acceptance. The AUC was 0.84, 0.94 and 0.92 for the models of pregnant women, non-pregnant women self-vaccination and child vaccination acceptance, respectively.

**Table 2:** Univariate analysis for predictors of COVID-19 vaccine acceptance among pregnant women and mothers of young children

Univariate analysis for acceptance of COVID-19 vaccine with ≥ 90% efficacy (likely vs. unlikely)	Accept for themselves				Accept for child/children	
	Pregnant women N = 5282		Non-pregnant mothers N = 12,562		All mothers N = 17,054	
Characteristics	Odds ratios	95% CI	Odds ratios	95% CI	Odds ratios	95% CI
Age (ref: 40–65 years)						
18–24	0.80	0.56, 1.14	0.66	0.55, 0.79	0.58	0.50, 0.66
25–29	0.88	0.63, 1.23	0.79	0.70, 0.89	0.71	0.64, 0.79
30–34	0.93	0.66, 1.30	0.94	0.85, 1.05	0.84	0.77, 0.93
35–39	0.92	0.65, 1.30	0.92	0.83, 1.03	0.86	0.78, 0.95
Married (ref: unmarried)	0.92	0.82, 1.03	1.10	1.01, 1.19	1.03	0.96, 1.10
Number of children (ref: no child)						
One child (used as ref for non-pregnant women only)	0.86	0.76, 0.97	NA	NA	0.91	0.81, 1.02
Two children	0.57	0.47, 0.68	0.79	0.72, 0.86	0.80	0.71, 0.90
Three or more children	0.43	0.33, 0.55	0.47	0.42, 0.52	0.50	0.45, 0.57
Education (ref: college diploma or equivalent)						
< High school	0.76	0.58, 0.99	0.97	0.82, 1.14	0.88	0.77, 1.00
High school or GED	1.25	1.03, 1.53	0.90	0.80, 1.02	0.96	0.87, 1.07
Some college or university	0.93	0.80 1.09	0.85	0.76, 0.95	0.85	0.78, 0.93
Master's, professional school, doctoral degree	1.26	1.10, 1.44	1.62	1.45, 1.81	1.54	1.41, 1.68
Socioeconomic status (SES)						
Middle class to wealthy (ref: lower middle class to poor)	0.92	0.82, 1.03	1.25	1.16, 1.36	1.14	1.06, 1.21
Essential workers (ref: non-essential workers)						
Physicians	2.26	1.54, 3.32	2.37	1.58, 3.56	2.03	1.52, 2.72
Nurses	0.71	0.57, 0.88	0.84	0.69, 1.03	0.75	0.65, 0.88
Other health workers	0.78	0.65, 0.94	0.76	0.64, 0.90	0.78	0.69, 0.89
Other essential workers (not health sector)	0.83	1.07, 1.23	0.84	0.75, 0.94	0.85	0.78, 0.94
Health status/insurance						
Have health insurance (ref: no health insurance)	0.93	0.81, 1.08	1.12	1.02, 1.22	1.10	1.02, 1.18
At least 1 underlying condition (ref: no underlying condition)	1.21	1.08, 1.35	1.40	1.29, 1.52	1.39	1.30, 1.49
Tested positive for COVID-19 (no COVID-19 test/ tested negative)	1.04	0.93, 1.17	1.26	1.15, 1.38	1.19	1.10, 1.28
Negative experiences with COVID-19						
Negatively affected by COVID-19 in any way (ref: not affected)	1.31	1.18, 1.47	1.32	1.22,1.43	1.31	1.23, 1.40
Suffered complications/disability due to COVID-19 disease (ref: no complications/disability)	1.97	0.85, 4.57	2.47	1.64, 3.74	2.14	1.51, 3.03
Lost a loved one to COVID-19 (ref: no loss)	2.82	2.03, 3.94	2.55	2.07, 3.13	2.63	2.20, 3.14
Other non-COVID-19 related health problems (ref: no additional health problems)	1.43	1.21, 1.68	1.37	1.25, 1.50	1.42	1.31, 1.54
Socially affected by COVID-19/restrictions (ref: not affected)	1.27	1.13, 1.42	1.26	1.17, 1.37	1.26	1.18, 1.34
Financial/job loss (ref: no loss)	1.16	1.02, 1.31	1.11	1.02, 1.20	1.11	1.04, 1.19
Past acceptance and perceived safety/efficacy of other vaccines						
Routine immunization for children (ref: no)	8.04	6.46, 10.0	21.2	18.1, 24.9	19.2	16.7, 22.0
Think childhood vaccines are safe (ref: not safe)	30.7	19.8, 47.7	44.6	36.4, 54.6	46.8	38.5, 56.8
Think childhood vaccines are effective in protecting against diseases (ref: not effective)	40.1	22.5, 71.3	48.1	37.9, 61.0	52.5	41.5, 66.4
Received flu vaccination last year or could not receive because of cost or availability (ref: did not receive for reasons other than cost or availability)	3.29	2.91, 3.72	5.24	4.81, 5.71	4.62	4.31, 4.95
Confidence in COVID-19 vaccine						
Confident in safety of COVID-19 vaccine post-approval by country's health agencies (ref: not confident)	8.42	7.44, 9.53	40.8	35.1, 47.5	27.8	25.0, 30.8
Confident in COVID-19 vaccine efficacy post approval by country's health agencies (ref: not confident)	6.68	5.90, 7.56	28.1	24.9, 35.6	19.7	18.1, 21.5
Perceived risk of the virus/precautions						
Likely to get COVID-19 (ref: unlikely)	1.45	1.29, 1.62	2.02	1.86, 2.19	1.80	1.68, 1.92
Likely for child to be infected with COVID-19 (ref: unlikely)	1.65	1.42, 1.93	1.88	1.73, 2.03	1.87	1.74, 2.00
Likely to develop severe symptoms if COVID-19 is contracted (ref: unlikely)	2.18	1.95, 2.43	2.97	2.73, 3.23	2.91	2.72, 3.12
Worried about COVID-19 (ref: not worried)	5.49	4.80, 6.28	12.2	11.1, 13.4	9.99	9.24, 10.8
Important for country to have vaccine (ref: not important)	24.1	17.7, 32.7	46.4	39.4, 54.7	45.0	38.7, 52.4
Important for the majority of people in their country getting vaccinated (ref: not important)	28.7	21.3, 38.6	52.0	45.0, 60.2	53.3	46.4, 61.2
Closely complied with mask guidelines (ref: did not comply)	13.7	9.23, 20.5	30.5	23.9, 39.0	24.9	20.2, 30.6
Monitoring COVID-19 news on any media once or less per day (ref: never)	3.56	2.88, 4.41	6.27	5.49, 7.17	5.91	5.26, 6.63
Public trust and satisfaction						
Trust in PH agencies (ref: no trust in PH agencies)	4.47	3.97, 5.03	13.2	11.9, 14.6	10.7	9.86, 11.5
Satisfied with the country's political leadership on COVID-19 control (ref: dissatisfied with political leadership)	1.08	0.96, 1.21	2.63	2.41, 2.89	2.13	1.98, 2.30
Trust in science (ref: no trust in science)	4.43	3.81, 5.15	7.91	7.21, 8.68	7.23	6.68, 7.83
Increased trust in science (ref: no increased trust in science)	3.94	3.50, 4.44	9.11	8.21, 10.1	7.42	6.85, 8.04
Satisfied with health authorities (ref: dissatisfied with health authorities)	1.68	1.50, 1.87	3.66	3.35, 4.01	3.06	2.85, 3.29
Satisfied with political response (ref: dissatisfied with political response)	1.62	1.44, 1.81	4.00	3.64, 4.40	3.32	3.08, 3.58

**Table 3:** Multivariate analysis for predictors of COVID-19 vaccine acceptance among pregnant women and mothers of young children, with adjustment for all countries

Multivariate analysis for acceptance of covid-19 vaccine with ≥ 90% efficacy (likely vs. unlikely)	Accept for themselves						Accept for child/children		
	Pregnant women N = 5282			Non-pregnant Mothers N = 12,562			All mothers N = 17,054		
	Adjusted odds ratio	95% CI		Adjusted odds ratio	95% CI		Adjusted odds ratio	95% CI	
Age (ref 40–65 years)									
18–24	1.20	0.73	1.99	0.93	0.67	1.28	0.96	0.75	1.22
25–29	1.04	0.66	1.66	0.92	0.74	1.14	0.83	0.70	0.99
30–34	0.90	0.57	1.42	1.02	0.85	1.22	0.86	0.74	1.00
35–39	0.83	0.52	1.33	1.02	0.85	1.22	0.85	0.73	0.99
Marital status									
Married (ref: not married)	1.03	0.86	1.23	1.03	0.89	1.20	0.96	0.85	1.08
Number of children (ref: no children)									
One child (used as ref for non-pregnant women only)	1.02	0.87	1.20	NA	NA	NA	0.90	076	1.08
Two children	0.92	0.72	1.18	0.94	0.81	1.10	1.00	0.82	1.20
Three or more children	1.12	0.77	1.63	0.78	0.64	0.94	0.92	0.74	1.15
Education (ref: college diploma or university degree)									
< High school	0.86	0.58	1.28	1.03	0.78	1.36	0.85	0.68	1.07
High school or GED	1.36	1.01	1.82	0.86	0.69	1.06	0.93	0.78	1.12
Some college or university	1.11	0.89	1.39	0.98	0.81	1.18	1.04	0.89	1.21
Master's, professional school, doctoral degree	0.79	0.66	0.94	1.01	0.84	1.21	0.90	0.79	1.04
Essential worker (ref: not an essential worker)									
Worked as a physician	1.48	0.93	2.35	0.88	0.47	1.63	0.73	0.49	1.10
Worked as a nurse	0.69	0.52	0.91	0.75	0.54	1.04	0.71	0.56	0.90
Worked in another health-related sector	0.79	0.62	1.00	0.84	0.62	1.12	0.80	0.65	0.99
Worked as another essential worker	0.99	0.81	1.21	1.00	0.83	1.20	1.05	0.90	1.21
Socioeconomic status (SES)									
Middle class to wealthy (ref: lower middle class to poor)	0.84	0.71	0.99	1.03	0.89	1.19	0.84	0.75	0.94
Insurance									
Insured (ref: uninsured)	0.95	0.77	1.18	1.20	1.03	1.41	1.22	1.07	1.39
Past acceptance and perceived safety/efficacy of other vaccines									
Routine immunization for children (ref: no)	1.75	1.27	2.41	2.26	1.68	3.05	2.42	1.90	3.08
Think childhood vaccines are safe (ref: not safe)	2.09	1.11	3.95	2.42	1.74	3.38	2.27	1.67	3.08
Thinks childhood vaccines are effective in protecting against diseases (ref: not effective)	1.57	0.69	3.58	1.48	1.01	2.18	1.39	0.97	1.99
Did receive flu vaccination last year (ref: did not receive)	1.40	1.18	1.67	1.58	1.36	1.82	1.45	1.29	1.63
Confidence in COVID-19 vaccine									
Confident in safety of COVID-19 vaccine post-approval by country's health agencies (ref: not confident)	3.68	3.02	4.50	5.12	4.06	6.46	4.97	4.23	5.84
Confident in COVID-19 vaccine efficacy post approval by country's health agencies (ref: not confident)	1.26	1.02	1.55	2.35	1.93	2.87	2.03	1.76	2.35
Perceived risk of the virus/precautions									
Worried about COVID-19 (ref: not worried)	1.79	1.48	2.16	2.47	2.11	2.90	1.99	1.75	2.26
Important for country to have vaccine (ref: not important)	1.74	1.05	2.87	1.68	1.25	2.25	1.60	1.22	2.09
Important for the majority of people in their country getting vaccinated (ref: not important)	3.08	1.95	4.85	4.46	3.50	5.68	4.89	3.91	6.12
Closely complied with mask guidelines (ref: did not closely comply)	1.10	0.65	1.89	2.69	1.80	4.04	1.85	1.32	2.57
Monitoring COVID-19 news on any media									
Monitors COVID-19 news at least 1 × a day (ref: never monitors)	1.17	0.86	1.59	1.06	0.83	1.35	1.21	0.99	1.48
Public trust and satisfaction									
Trust in PH Agencies (ref: no trust in PH agencies)	1.14	0.95	1.35	1.62	1.38	1.89	1.51	1.34	1.70
Trust in science (ref: no trust in science)	1.21	0.97	1.51	1.16	0.99	1.37	1.24	1.09	1.42
Increased trust in science during COVID-19 (ref: no increased trust in science)	1.31	1.12	1.54	1.33	1.13	1.56	1.26	1.12	1.43

These findings persist in all within-country analyses (Table 2S), so we present the pooled analysis only. The correlation between the top predictors and vaccine acceptance found at the individual level is also reflected at the ecologic level (e.g., countries with higher trust in public health agencies tend to have higher acceptance).

## Interpretations

We found substantial geographic variation in the acceptance of COVID-19 vaccination among pregnant women and mothers of young. Acceptance in India, the Philippines, and Latin America was above 60% among pregnant women and above 78% among non-pregnant women for themselves; and above 75% among mothers for their children. This is in contrast to women in the US and Russia, who consistently expressed lower acceptance, confidence in COVID-19 vaccine safety/effectiveness, perceived importance of COVID-19 vaccination as well as public trust. Confidence in beneficial vaccine outcomes and trust of public health agencies was particularly low in Russia, compared to the other 15 countries in this study. Women’s risk perception of COVID-19 in the US and Russia, two countries, was comparable to that in low incidence countries (i.e. Australia and New Zealand), potentially suggesting a phenomenon of COVID-19 denial. These results underscore that a high burden of disease alone may not provide sufficient motivation for pregnant women and mothers of young children to seek vaccination for themselves or their children.

Country-level observations were largely confirmed with within-country analysis of individual responses. The strongest predictors of COVID-19 vaccine acceptance were confidence in COVID-19 vaccine safety and effectiveness, belief in the importance of vaccines/mass vaccination to their own country, confidence in routine childhood vaccines, worry about COVID-19, trust of public health agencies and science, as well as compliance with face mask-wearing guidelines. Although this may suggest overlap in general vaccine confidence, it should be noted that skepticism of COVID-19 vaccine safety and effectiveness was far greater within all countries. The International Registry of Coronavirus Exposure in Pregnancy (IRCEP) and several safety surveillance systems are underway to collect such information [21]. The results of these efforts may influence several of the significant factors identified here, providing more specific data for these populations, and opportunities to build on trust in the scientific review of such vaccines.

Our findings of the heterogeneous COVID-19 vaccine acceptance level among pregnant women and mothers in different countries are consistent with previous reports from the general population. Polls conducted in the US in July 2020 showed that only 66% of adults would be likely to vaccinate themselves or their children [10]. In a global survey conducted in June 2020 in 19 countries, 71.5% of participants stated that they would be likely to get vaccinated for COVID-19, but acceptance levels ranged from 90% in China to less than 55% in Russia. A relatively high likelihood of acceptance in middle-income countries, such as Brazil, India and South Africa, was also reported [11]. The higher acceptance among the middle-income countries suggests the role historical burdens of other infectious diseases might play on both the higher perception of risk from COVID-19 and the more positive vaccine attitude.

These results confirmed our hypothesis that COVID-19 vaccine hesitancy is a multifaceted event [22]. Perceived risk of the virus and the disease, as well as public trust, play key roles shaping the vaccine acceptance and confidence on top of the existing pre-COVID 19 vaccine attitudes. Perceived risk of COVID-19 for the population, as measured by worries of COVID-19, belief in importance of having a COVID-19 vaccine and mass vaccination, was a much stronger predictor of COVID-19 vaccine acceptance and confidence in comparison to perceived risk of infection for themselves or their children. Doubt of the disease, skepticism of a new vaccine, distrust of the system, along with previously shaped vaccination belief have interplayed and together contributed to the reluctance we observed. Even though these parameters are dynamic and being influenced by current events in this pandemic, their roles do seem to follow what we have known in the past regarding the general factors for vaccine hesitancy [22–26].

Our findings suggest that there is room for governments, especially those of countries currently experiencing COVID-19 denial and public distrust to rebuild confidence in the coming months as vaccine roll-out continues. Policy makers must address such denial and doubt of the disease, public fears and misconception using clear and unified communication to create a national consensus of the utter importance of public health measures including face mask-wearing and mass vaccination to end this pandemic crisis. The public risk perception and confidence of a new vaccine can thus be put into perspectives of what is truly at stake. Health providers’ recommendation alone will have limited effects on improving acceptance among this population. For those who do not follow COVID-19 news through any media and have high vaccine reluctance, alternative communication methods are needed. Lessons learned from previous new vaccine roll-out including human papillomavirus (HPV) vaccine roll-out and Ebola vaccine trial field experiences have all underscored the importance of community work with sensitivity toward country-, local- and subgroup- specific culture contexts [27, 28]. Significant portions of pregnant women and mothers expressed additional safety concerns due to insufficient pregnancy- and children- related clinical evidence. As more such data become available for these two groups, there will be more opportunities for pregnant women and mothers to build trust on the scientific approval of these vaccines.

This survey captured rich data in a large study population from a wide span of COVID-19-affected countries on the topics of vaccine acceptance and its predictors. Findings provide important information that can be used for evidence-based policy making to ultimately enhance vaccination of vulnerable populations such as pregnant women and children. However, there are several study limitations to note. Although the survey encompasses responses from 16 countries and was available in six languages, the responses may not be generalizable to other regions. However, the consistency among the 16 geographically disperse countries strongly suggest that predictors are likely to be universal. Selection bias is a possibility as participants had an overall higher education level than the general public and were predominantly white in the US and European countries. Furthermore, as the survey was conducted online, study participants were limited to those who had access to the technology and resources to participate in the survey. This limitation could have prevented vaccine acceptance levels captured from the most vulnerable groups with generally higher than average levels of vaccine hesitancy and caused an overall overestimation of the acceptance. The overall sample was not probability-weighted based on each country’s population size, and therefore results should not be viewed as weighted-average estimates from all the 16 countries’ populations of women of child-bearing age. Our intention was rather to understand the most important predictors and the underlying interplay between the factors and to draw key insights for global policymakers. Like all online surveys, responses differ based on the respondent’s personal attitudes, feelings, and inherent biases, and are therefore a snapshot of the vaccine acceptance in time of a highly dynamic and evolving landscape in these countries. Vaccine acceptance levels might change over the course of time, especially as vaccine education campaigns begin in these countries.

In the two months since this study was conducted, several important events have occurred: an alarming surge in new coronavirus infections and deaths worldwide, the authorization of several COVID-19 vaccines, with messenger ribonucleic acid (mRNA) vaccines achieving efficacies as high as 95%, and the initiation of vaccination campaigns with millions of doses being administered globally [1, 4–6, 29].These developments will likely impact the key predictors such as COVID-19 risk perception, confidence in the COVID-19 vaccine and public trust and may improve public acceptance, as suggested by more recent national surveys in the US [30]. As global vaccine rollouts continue, monitoring acceptance and its predictors will provide policymakers with key insights to understand public willingness and make informed policies that are effective. It is especially urgent for countries currently experiencing widespread public distrust during the pandemic to rebuild vaccine confidence through transparent communication and effective community engagement. COVID-19 vaccine education campaigns need to emphasize the pandemic as a whole and what’s at stake for communities, instead of a limited focus of vaccine safety and effectiveness. As more specific data for these vulnerable groups become available, it will provide opportunities to influence key predictors we identified and enhance public trust in the rigorous approval of upcoming vaccines [21].

Current acceptance levels of a COVID-19 vaccine among most of the high-income countries included in this study are insufficient to meet the requirements for community immunity of at least 75%. COVID-19 vaccine acceptance and its predictors among pregnant women and mothers of young children vary globally and, therefore, vaccination campaigns for this population should be specified for each country in order to attain the largest impact.

## Supplementary Information

Below is the link to the electronic supplementary material.

Supplementary file1 (PDF 1982 KB)

## Data Availability

Data for this study will be made available to others in the scientific community upon request one year after the publication of this article. Standard criteria for making data available for valid research projects will be used, following application by suitably qualified researchers. For data access, please contact Pregistry at hello@pregistry.com.

## Abbreviations

COVID-19: Coronavirus disease 2019
IRCEP: International registry of coronavirus exposure in pregnancy
GED: General equivalent degree
HCP: Health care provider
HPV: Human papillomavirus
mRNA: Messenger ribonucleic acid
SARS-CoV-2: Severe acute respiratory syndrome coronavirus 2ß
UK: United Kingdom
US: United States of America
WHO: World Health Organization

## Code availability

The code for this study will be made available to others in the scientific community upon request one year after the publication of this article. Standard criteria for making the code available for valid research projects will be used, following application by suitably qualified researchers. For code access, please contact Julia Wu.
