# Supplementary material for: COVID-19 vaccine acceptance among pregnant women and mothers of young children: results of a survey in 16 countries
Source: Eur J Epidemiol. 2021 Mar 1;36(2):197–211. doi: 10.1007/s10654-021-00728-6 (PMC7920402; doi:10.1007/s10654-021-00728-6)
Supplement: Supplementary file 1 — Supplementary file1 (PDF 1982 KB) [file 10654_2021_728_MOESM1_ESM.pdf]

Supplemental Information

COVID-19 Vaccine Acceptance Among Pregnant Women and Mothers of Young Children: Results of a Survey in 16 Countries

Malia Skjefte[1], Michelle Ngirbabul[1], Oluwasefunmi Akeju, MBChB, MPH[2], Daniel Escudero, PhD, MPH[2], Sonia Hernandez-Diaz, MD, MPH, DrPH[2], Diego Wyszynski, MD, MHS, PhD[3], Julia W Wu, ScD[2*]

[1]Department of Global Health & Population, Harvard TH Chan School of Public Health, Boston, MA, USA
[2]Department of Epidemiology, Harvard TH Chan School of Public Health, Boston, MA, USA
[3]Pregistry, Los Angeles, CA, USA

*Correspondence to:
Julia W Wu, ScD
Human Immunomics Initiative
Department of Epidemiology
Harvard TH Chan School of Public Health
Boston, MA 02459, USA
wwu@hsph.harvard.edu

Survey Questionnaire

The survey tool contained 63 questions divided into five sections, assessing COVID-19 vaccine acceptance and confidence, negative experiences with COVID-19, perception on the risk of COVID-19, public trust, general vaccine attitude, as well as demographics and socioeconomic status. Questions included a mixture of open ended-questions, dichotomous yes/no questions, and multiple-choice questions. Responses to multiple-choice questions measured agreement with 5- or 7-point Likert scales. Section One collected basic participant demographics, including age, race, marital status, number of children, and pregnancy status. Section Two collected information regarding COVID-19, such as exposure, essential worker status, health insurance, underlying health conditions, how worried they are about the virus, their own risk, and the risk to their child/children. Section Three assessed the impact of COVID-19 pandemic, including social, financial, loss of a loved one, or non-COVID-19 related health problems. Section Four assessed the respondents' vaccine acceptance, vaccine confidence, and factors influencing decision-making. Section Five assessed social cohesion, trust, and satisfaction. Participant responses were submitted directly through the website, and no personal identifiers were collected. Participants were allowed to skip any questions or withdraw from the survey at any time without penalty. No follow-up was conducted.

Figure S1: Importance of a COVID-19 vaccine among global pregnant women and mothers of young children

a) Importance for the country where respondents currently live to have a COVID-19 vaccine (n=17769)

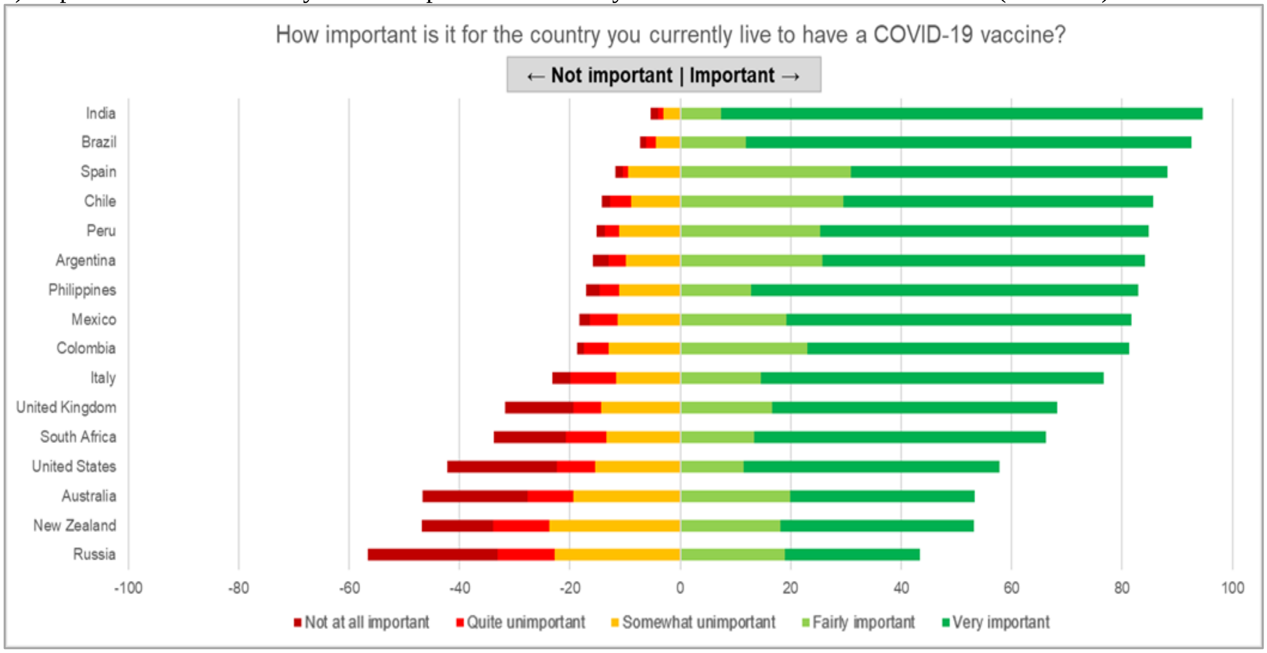

b) Importance for a majority of people in the country where respondents currently live to get vaccinated for COVID-19 (n=17761)

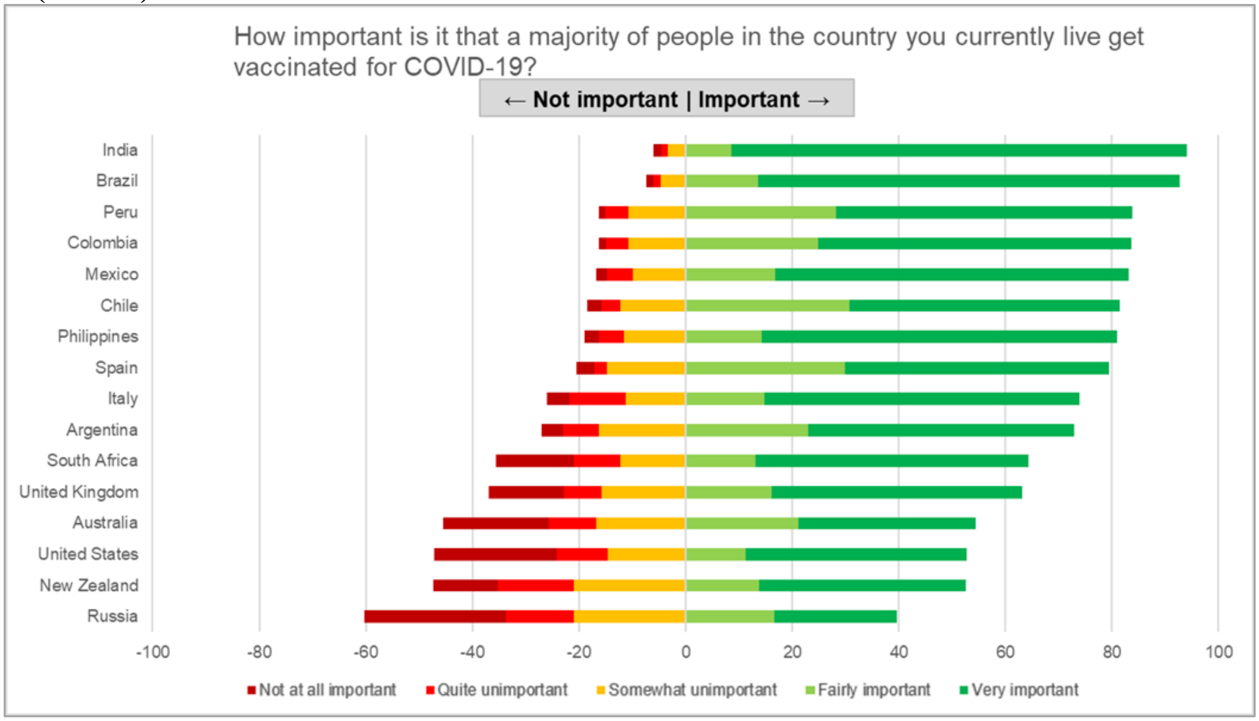

Figure S2: Trust in science among global pregnant women and mothers of young children

a) Respondents' general trust in health science (n=17837)

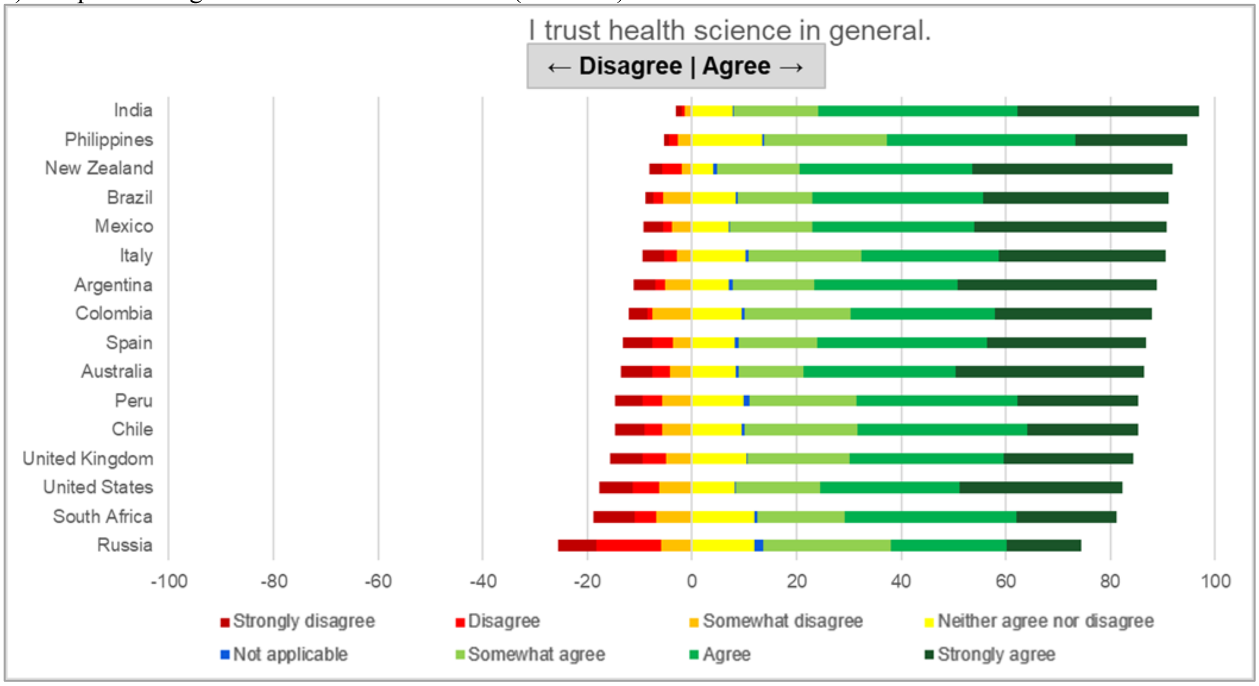

b) Respondents' general increased trust in health science during the COVID-19 pandemic (n=17837)

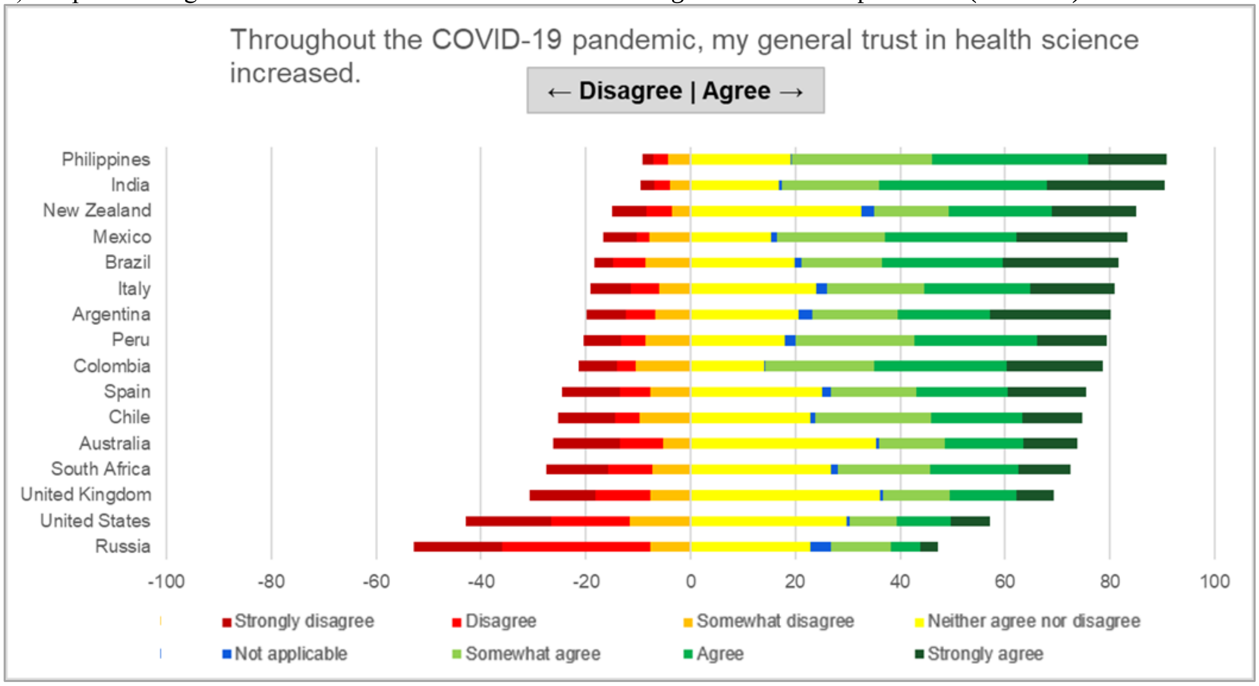

Figure S3: Satisfaction with COVID-19 control among global pregnant women and mothers of young children

a) Satisfaction with medical scientists for their performance in controlling COVID-19 in their countries (n= 17837)

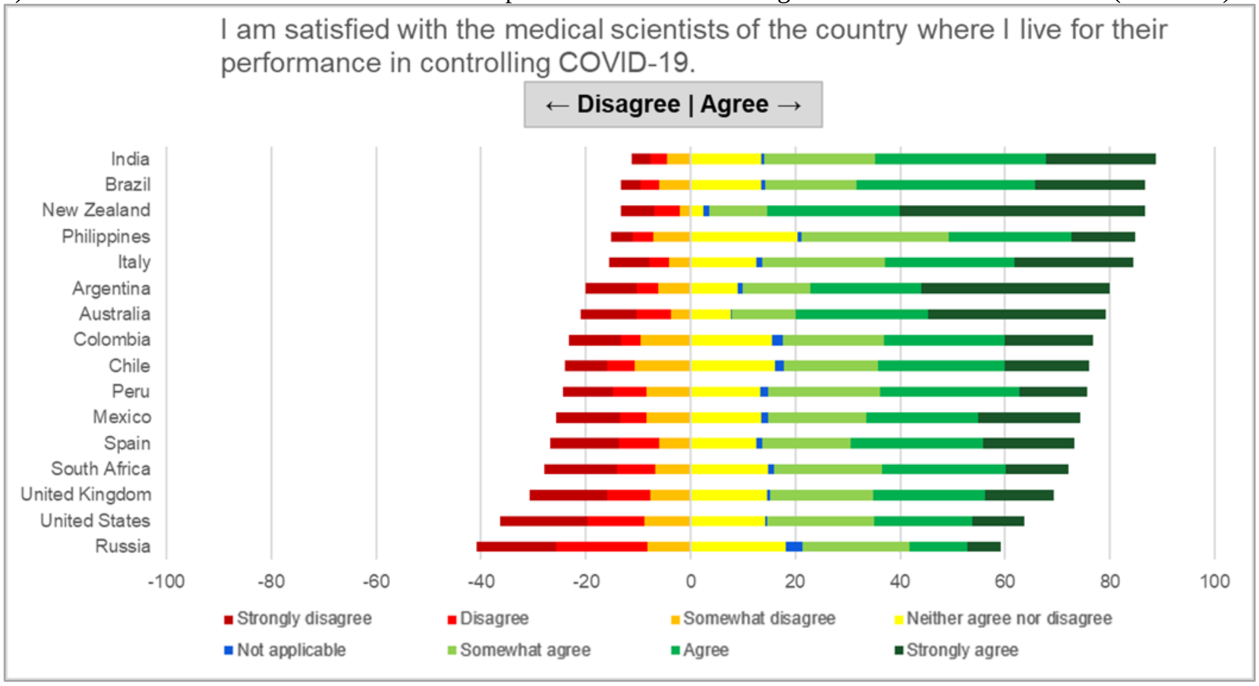

b) Satisfaction with health authorities for their performance in controlling COVID-19 in their countries (n= 17837)

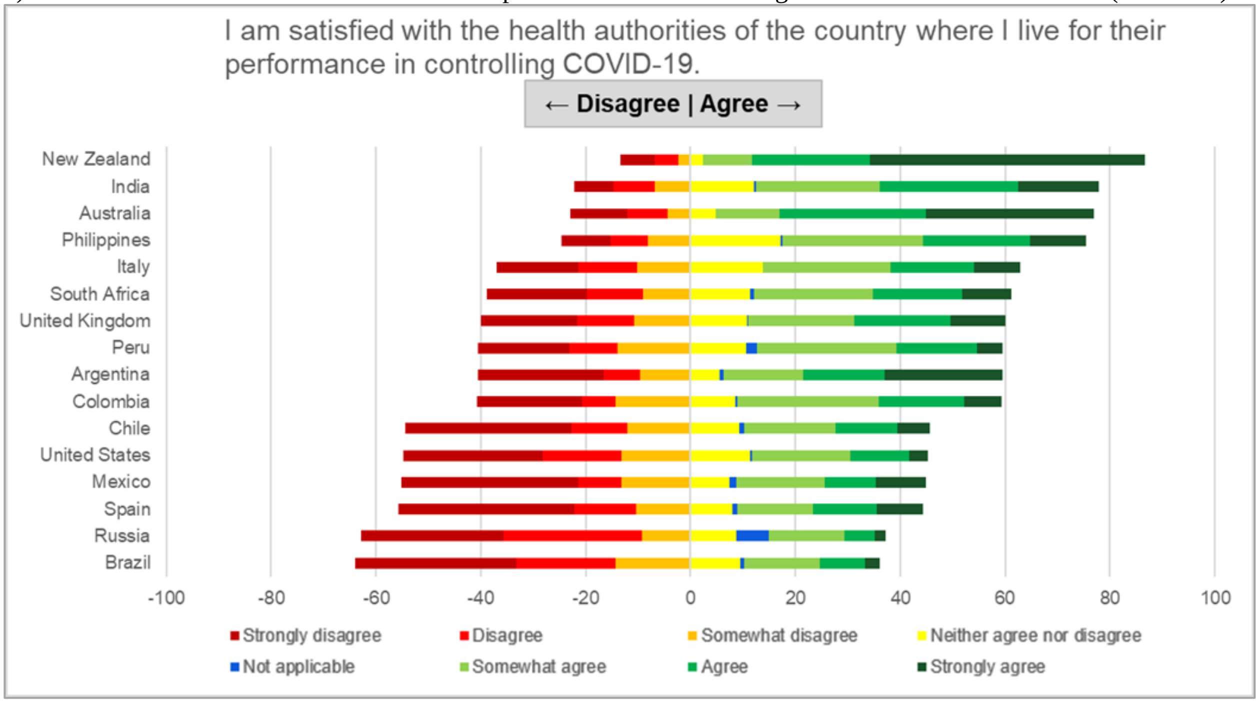

Figure S4: Information COVID-19 and vaccine development among global pregnant women and mothers of young children

a) Respondents' feelings of how informed they are about COVID-19 and vaccines being developed (n=17747)

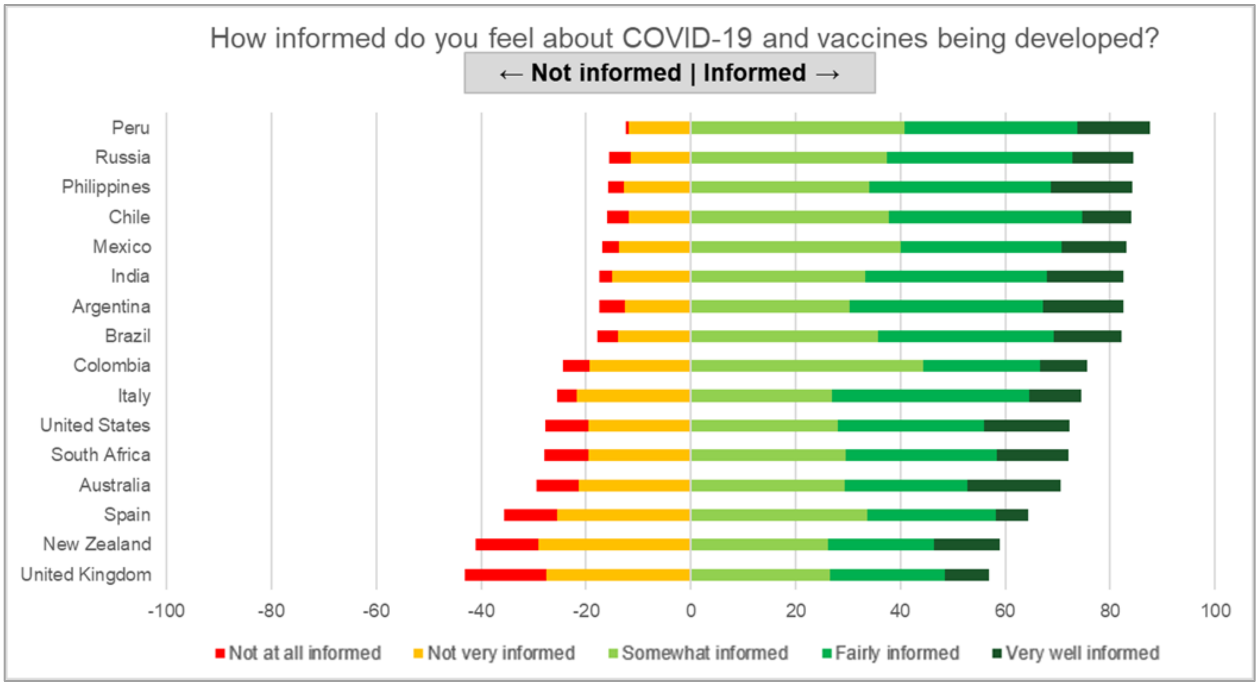

Figure S5: Perceptions on childhood vaccines among global pregnant women and mothers of young children

a) Perceptions on safety of childhood vaccines (n=17840)

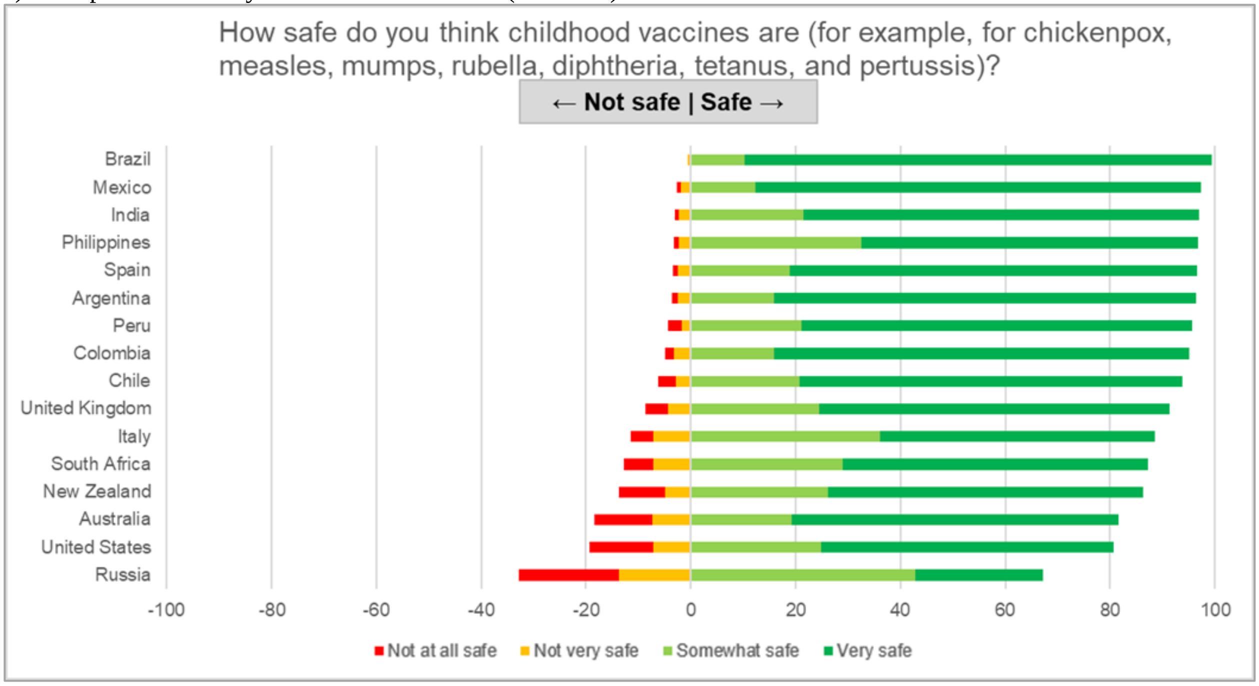

b) Perceptions on effectiveness of childhood vaccines (n=17839)

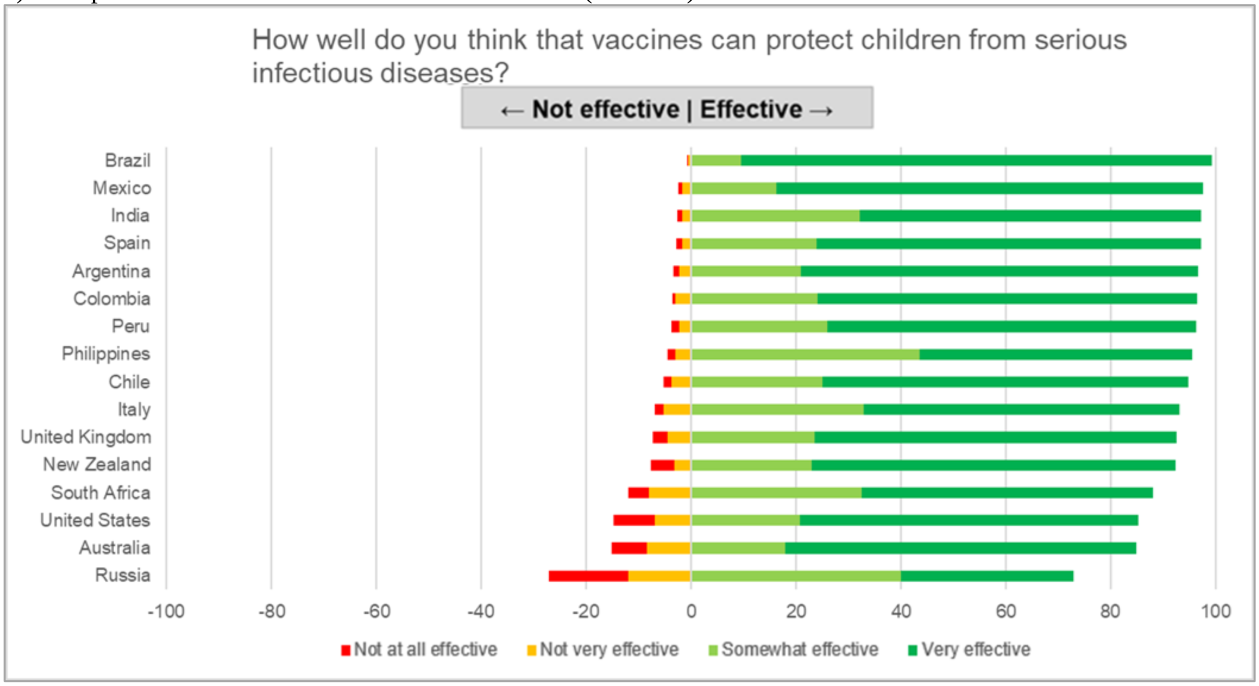

Figure S6: Key predictors of vaccine acceptance among pregnant women, non-pregnant women, and mothers/mothers-to-be

a) Confidence in COVID-19 vaccine safety

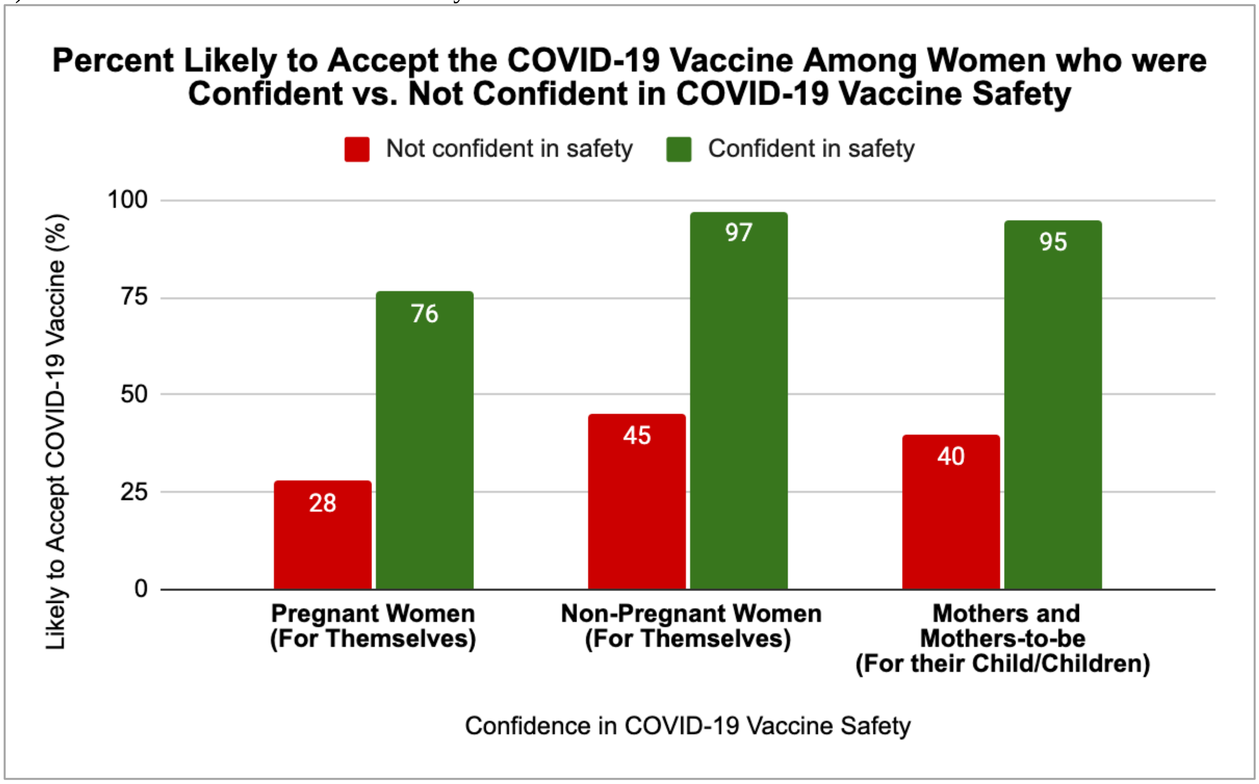

b) Confidence in COVID-19 vaccine efficacy

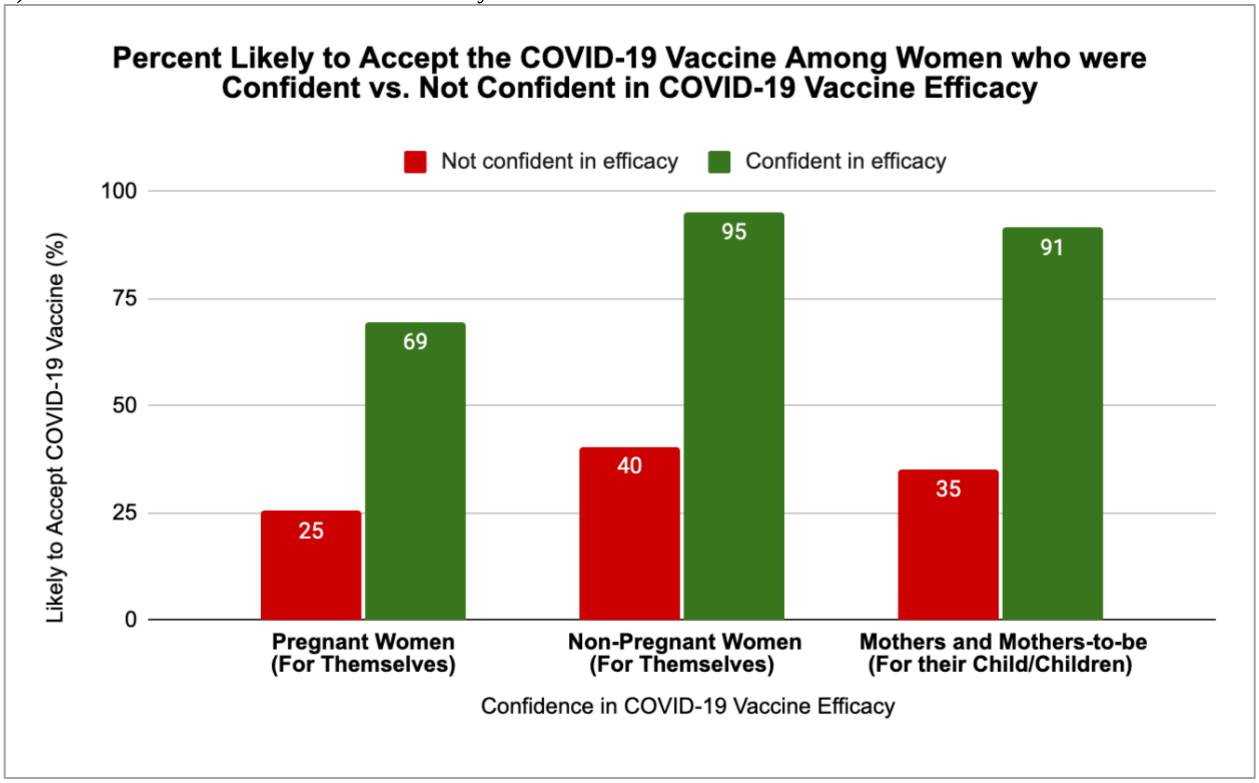

c) Worry regarding COVID-19

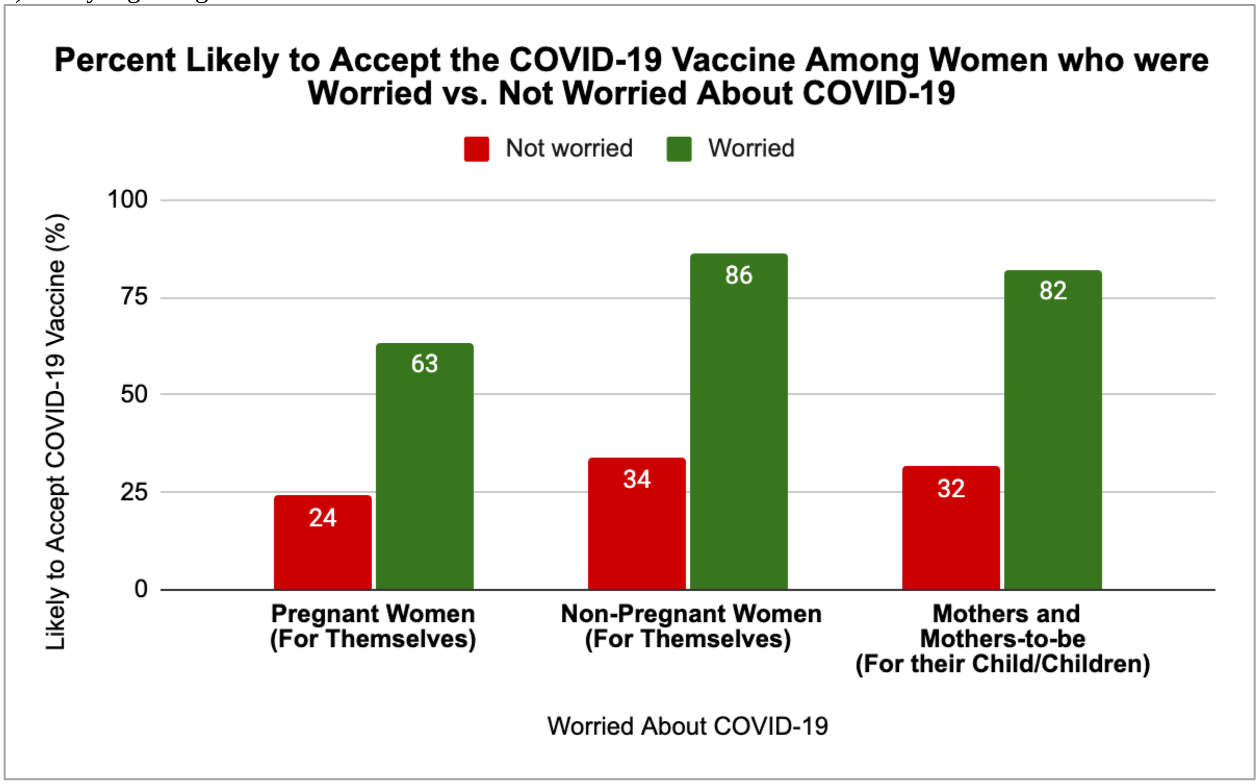

d) Trust in public health agencies

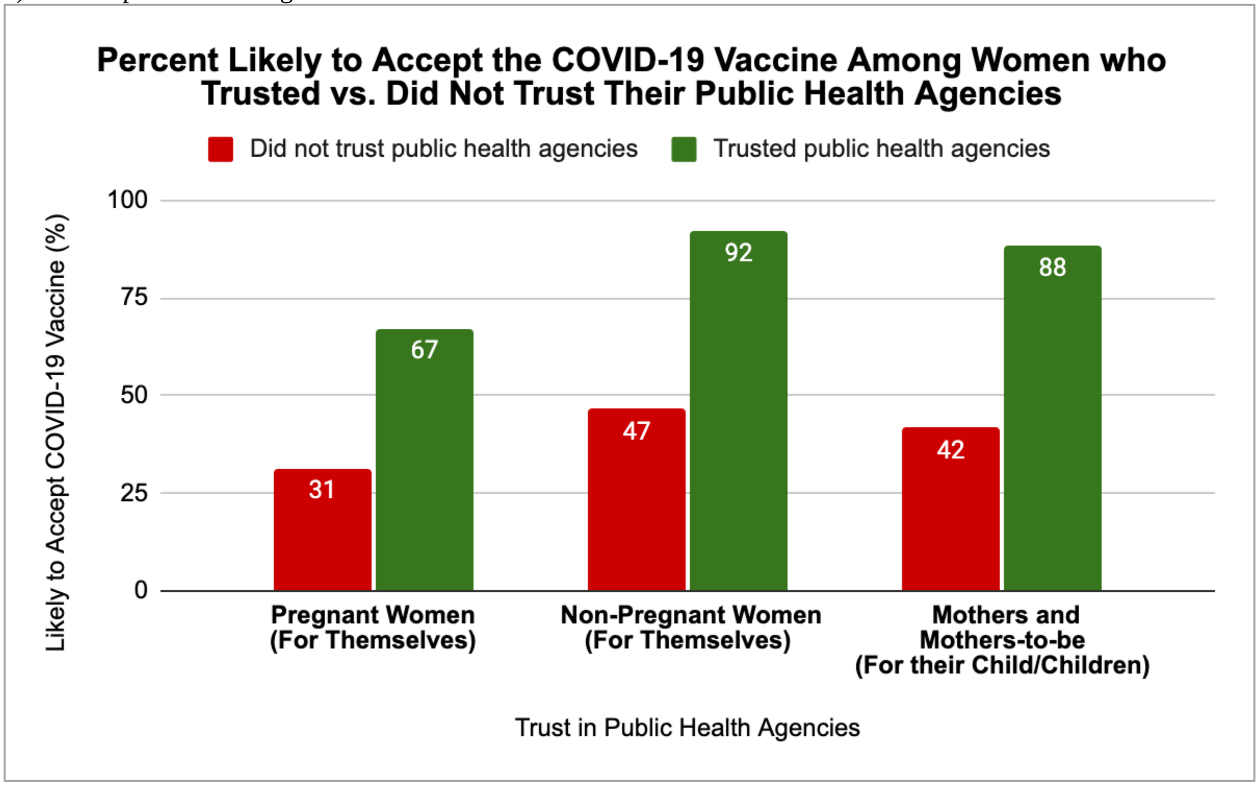

e) Trust in health science

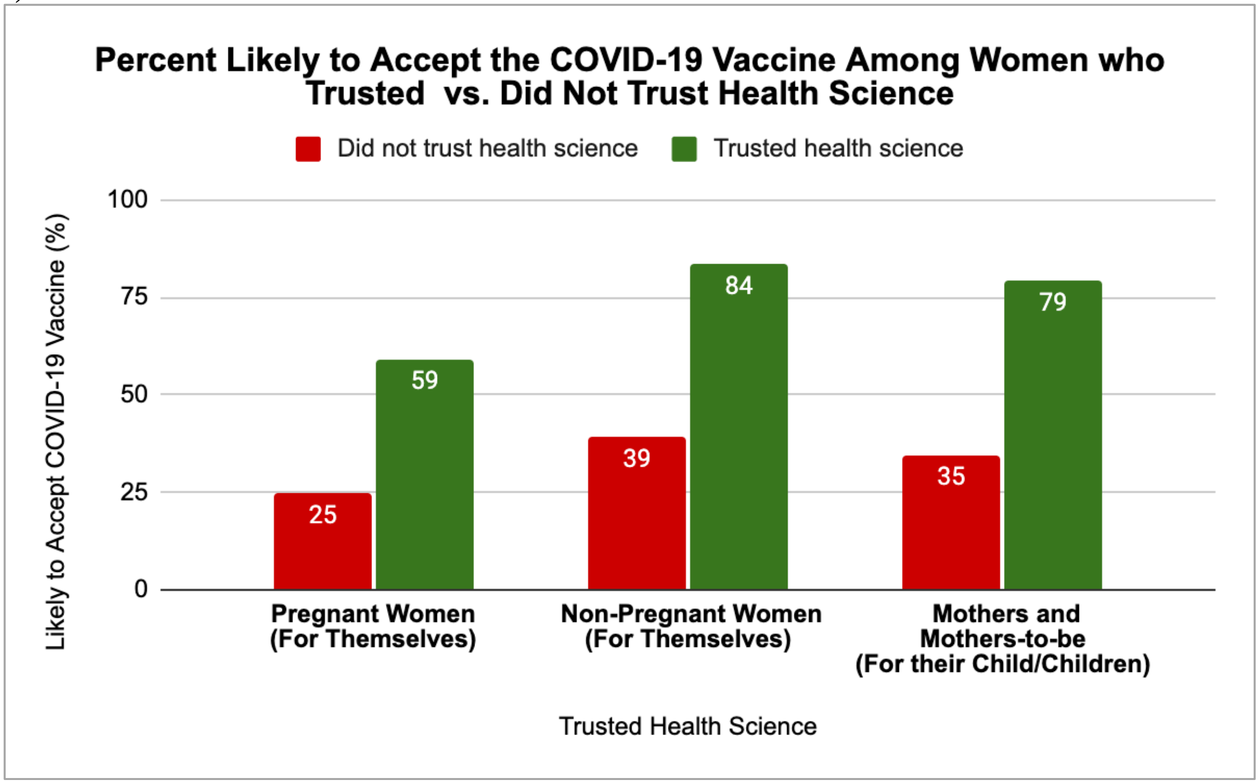

f) Confidence in safety of routine childhood vaccines

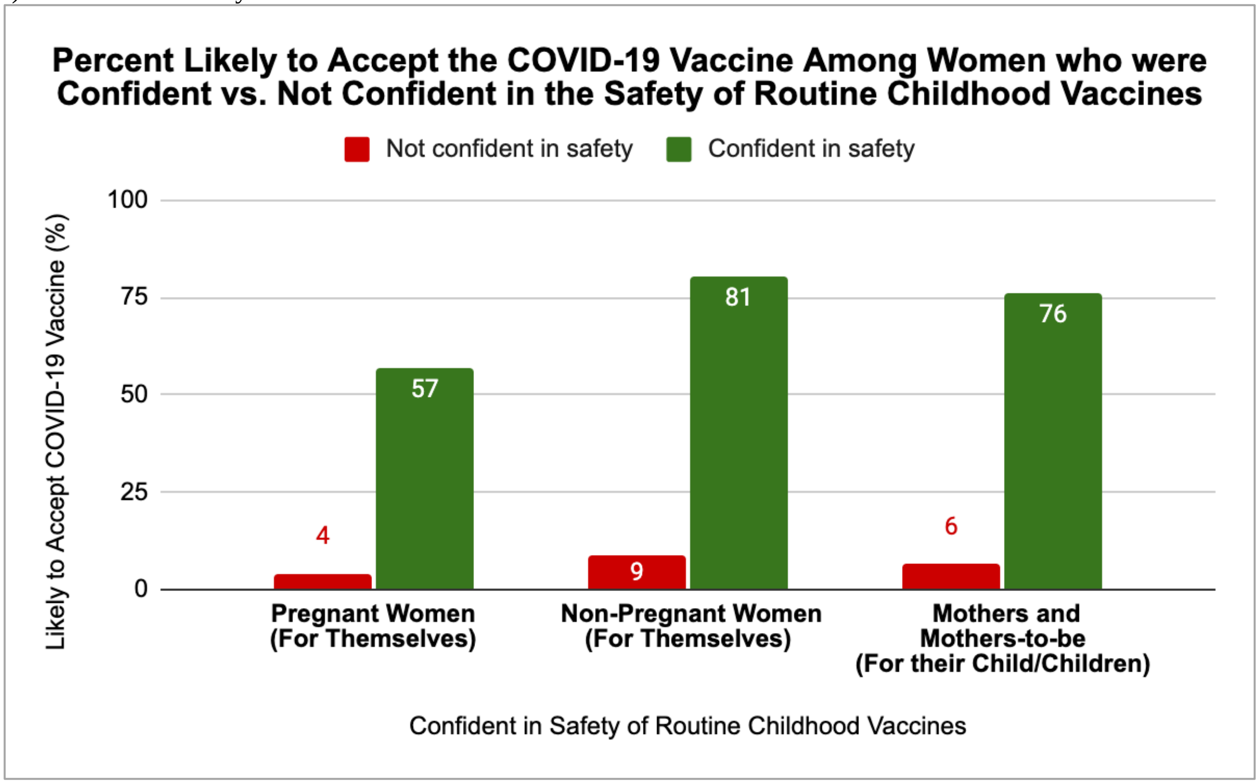

g) Confidence in efficacy of routine childhood vaccines

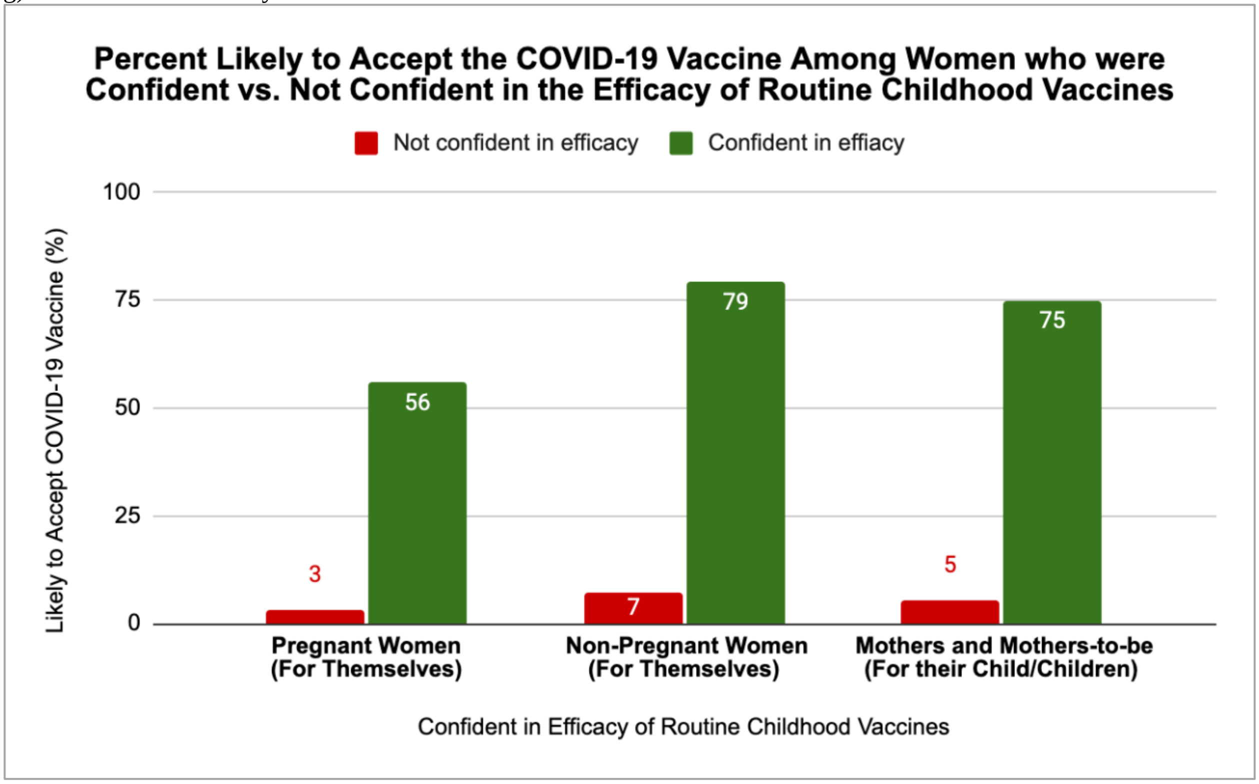

h) Belief in the importance for their country to have a COVID-19 vaccine

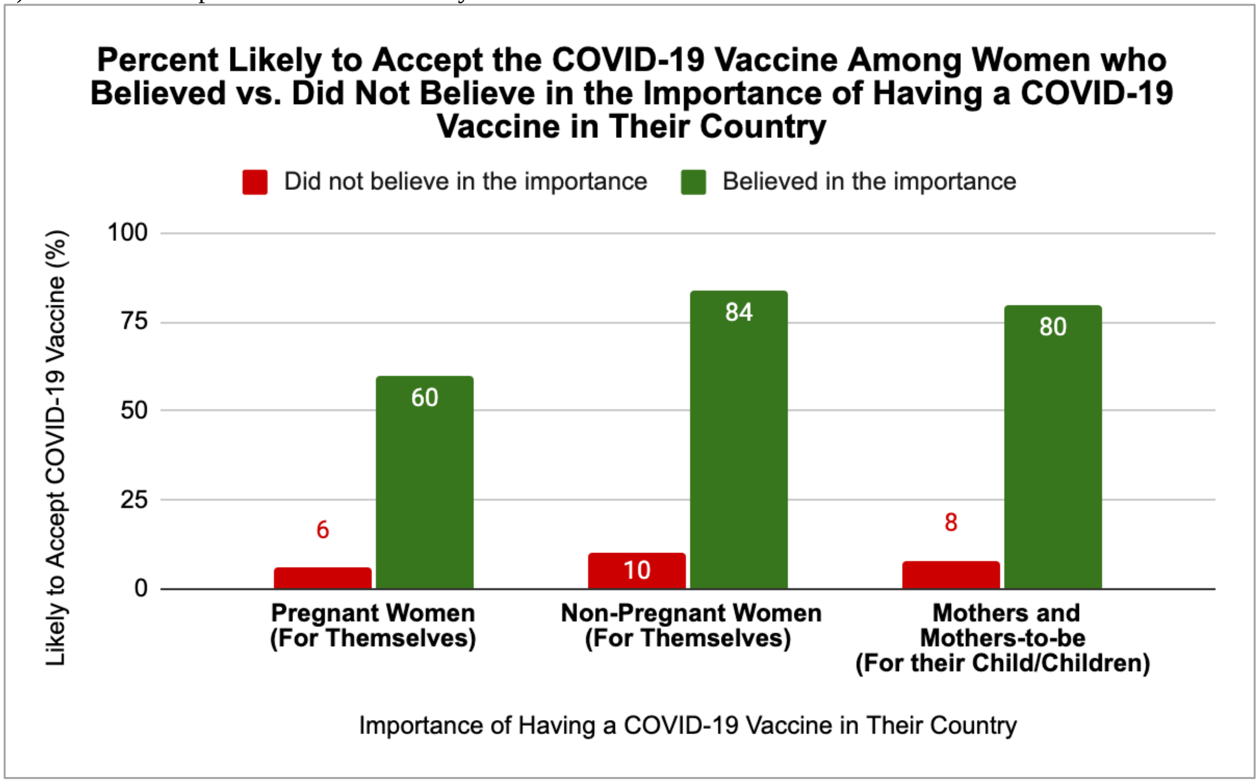

i) Belief in the importance of mass COVID-19 vaccination in their own country

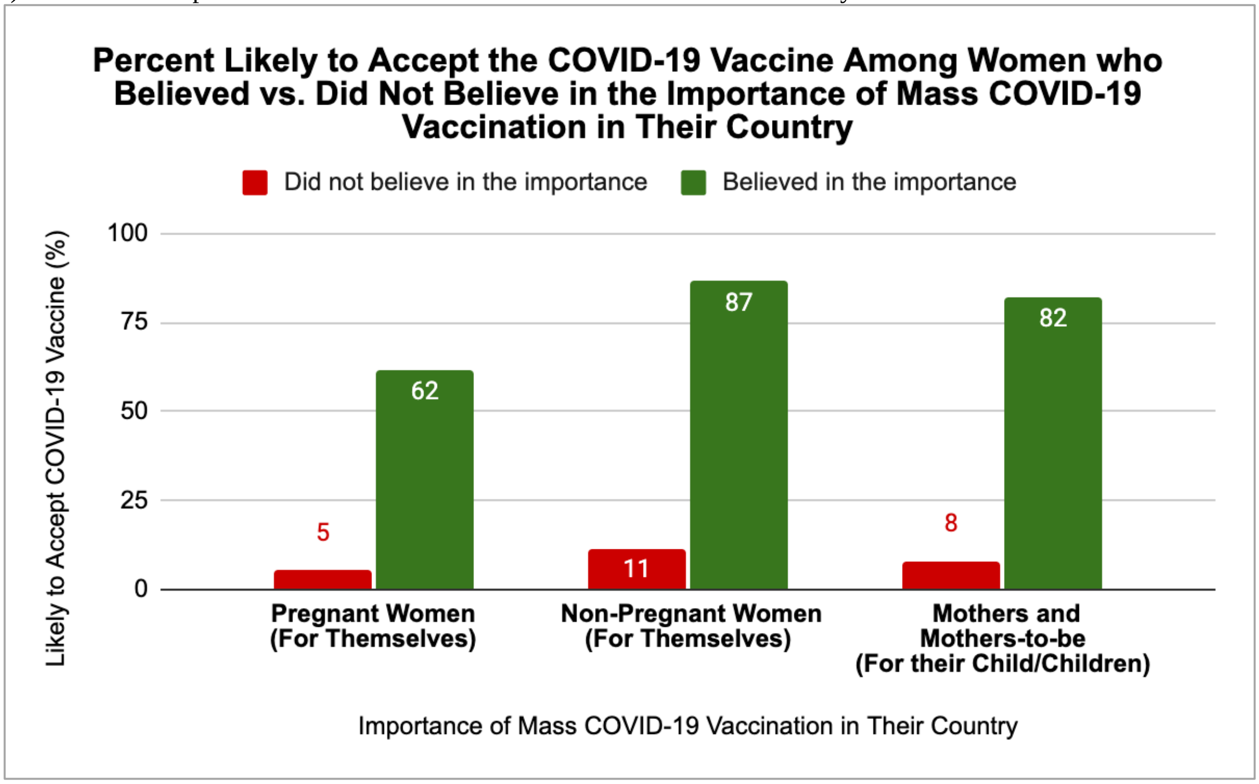

j) Compliance with face mask wearing guidelines

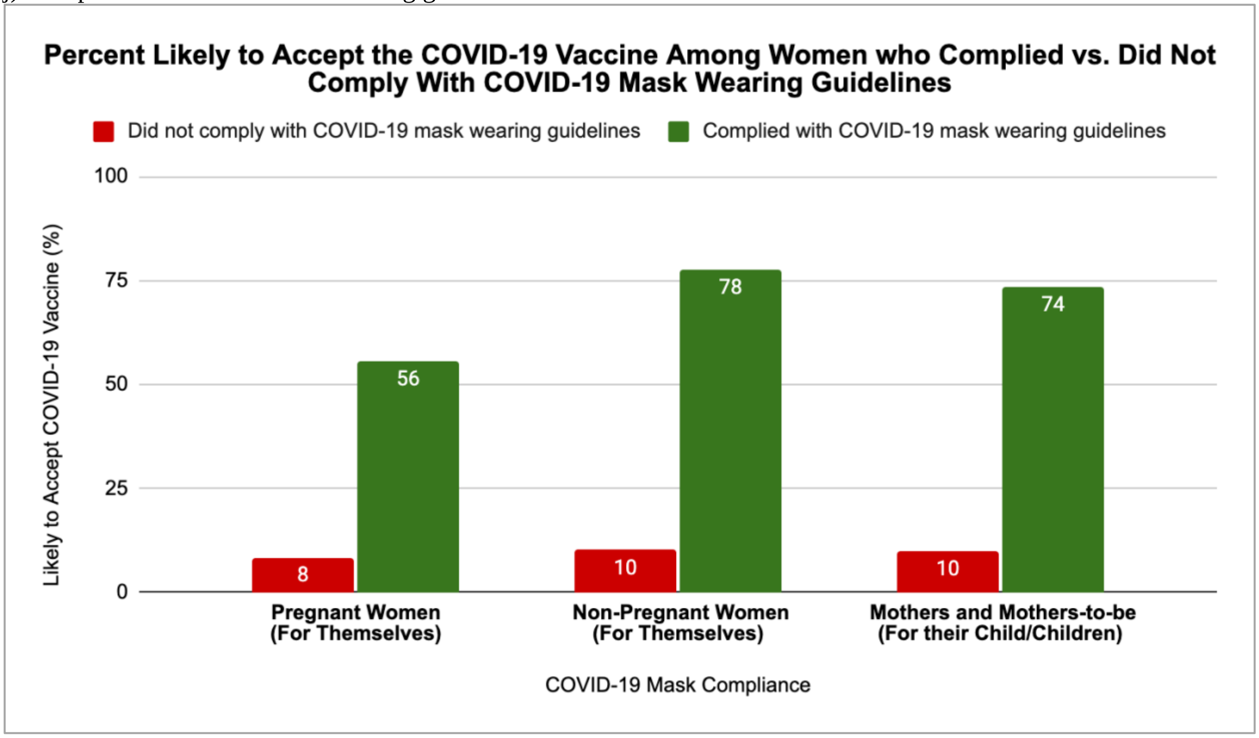

k) Monitoring of COVID-19 news and media

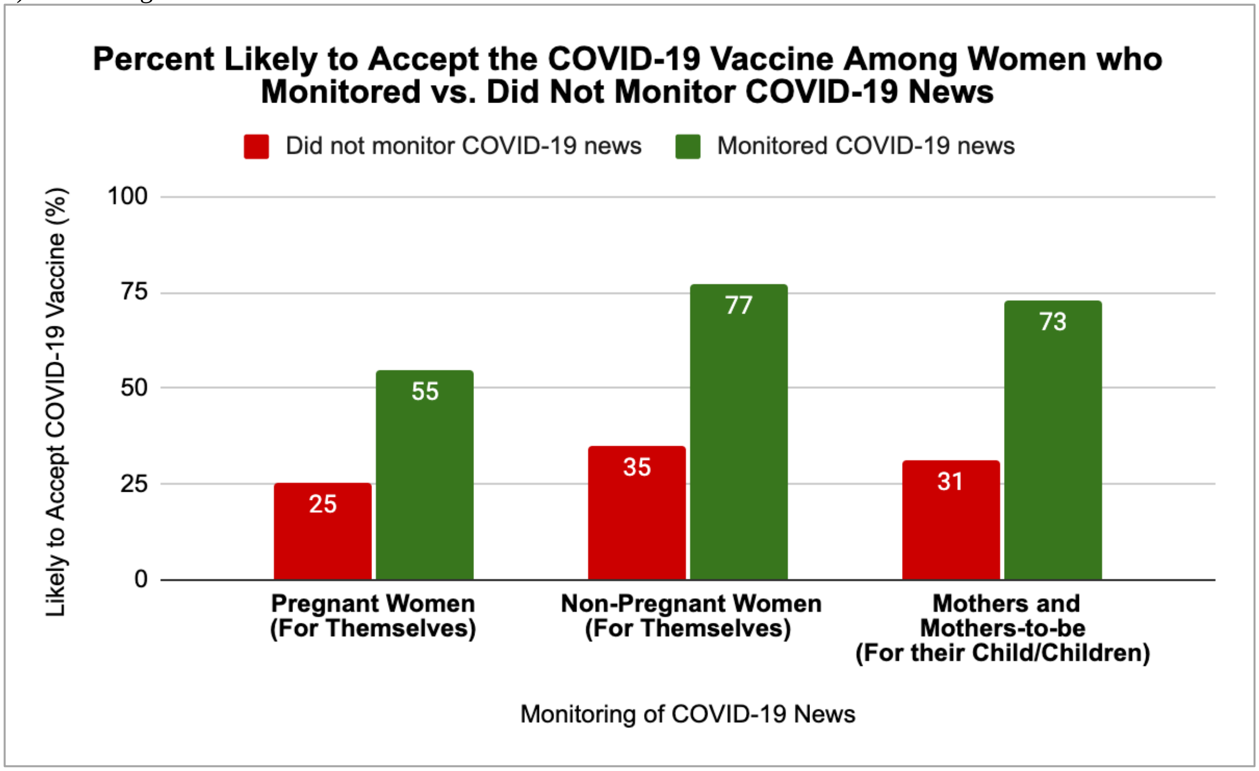

Table 1S: Complete Demographics for 16 Countries (n=17871)

	Overall	North America		South America					Africa	Europe				Asia		Oceania	
		United States	Mexico	Brazil	Colombia	Chile	Argentina	Peru	South Africa	Spain	Italy	Russia	United Kingdom	Philippines	India	Australia	New Zealand
**n (%)**	***17871 (100)**	**4014 (22·5)**	**1005 (5·6)**	**932 (5·2)**	**516 (2·9)**	**434 (2·4)**	**1632 (9·1)**	**347 (1·9)**	**686 (3·8)**	**672 (3·8)**	**995 (5·6)**	**537 (3·0)**	**2702 (15·1)**	**1034 (5·8)**	**1639 (9·2)**	**476 (2·7)**	**250 (1·4)**
Pregnant (%)																	
Yes	5294 (29·6)	2010 (50·1)	167 (16·6)	631 (67·9)	48 (9·3)	74 (17·1)	205 (12·6)	34 (9·8)	69 (10·1)	187 (27·9)	236 (23·7)	154 (28·7)	790 (29·3)	91 (8·8)	253 (15·4)	256 (53·8)	89 (35·6)
No	12571 (70·4)	2004 (49·9)	837 (83·4)	299 (32·2)	468 (90·7)	360 (83·0)	1427 (87·4)	312 (90·2)	617 (89·9)	484 (72·1)	759 (76·3)	383 (71·3)	1,911 (70·8)	943 (91·2)	1386 (84·6)	220 (46·2)	161 (64·4)
Children (%)																	
Yes	15198 (85·1)	3,095 (77·1)	911 (90·7)	567 (61·0)	487 (94·4)	407 (93·8)	1525 (93·6)	328 (94·8)	655 (95·5)	567 (84·5)	888 (89·3)	485 (90·5)	2276 (84·3)	1005 (97·2)	1464 (89·3)	334 (70·3)	205 (81·9)
No	2661 (14·9)	919 (22·9)	93 (9·3)	363 (39·0)	29 (5·6)	27 (6·2)	104 (6·4)	18 (5·2)	31 (4·5)	104 (15·5)	107 (10·8)	51 (9·5)	425 (15·7)	29 (2·8)	175 (10·7)	141 (29·7)	45 (18·1)
No. Children (%)																	
0	2661 (14·9)	919 (22·9)	93 (9·3)	363 (39·0)	29 (5·6)	27 (6·2)	104 (6·4)	18 (5·2)	31 (4·5)	104 (15·5)	107 (10·8)	51 (9·51)	425 (15·7)	29 (2·8)	175 (10·7)	141 (29·7)	45 (18·1)
1	6795 (38·1)	1243 (31·0)	401 (39·9)	331 (35·6)	249 (48·3)	170 (39·2)	577 (35·4)	143 (41·3)	230 (33·5)	288 (42·9)	482 (48·4)	231 (43·1)	910 (33·7)	363 (35·1)	950 (58·0)	147 (31·0)	80 (32·1)
2	5465 (30·6)	1004 (25·0)	375 (37·4)	168 (18·1)	191 (37·0)	150 (34·6)	564 (34·6)	122 (35·3)	285 (41·6)	217 (32·3)	316 (31·8)	198 (36·94)	862 (31·9)	344 (33·3)	489 (29·8)	102 (21·5)	78 (31·3)
3	1948 (10·9)	499 (12·4)	106 (10·6)	46 (5·0)	38 (7·4)	63 (14·5)	264 (16·2)	53 (15·3)	104 (15·2)	50 (7·5)	70 (7·0)	44 (8·21)	321 (11·9)	188 (18·2)	20 (1·2)	49 (10·3)	33 (13·3)
4+	990 (5·5)	349 (8·7)	29 (2·9)	22 (2·4)	9 (1·7)	24 (5·5)	120 (7·4)	10 (2·9)	36 (5·3)	12 (1·8)	20 (2·0)	12 (2·24)	183 (6·8)	110 (10·6)	5 (0·3)	36 (7·6)	13 (5·2)
Age (%)																	
Mean (sd)	34·44 (7·3)	32·15 (6·4)	34·96 (7·6)	31·10 (6·2)	35·11 (7·6)	36·57 (7·5)	36·79 (7·6)	35·96 (8·4)	36·99 (8·0)	36·02 (7·1)	36·84 (7·0)	34·67 (6·4)	35·41 (7·5)	35·1 (7·9)	33·59 (5·83)	32·46 (6·7)	34·08 (8·0)
18-24	1299 (7·3)	420 (10·5)	91 (9·1)	142 (15·3)	41 (8·0)	23 (5·3)	86 (5·3)	29 (8·4)	51 (7·4)	30 (4·5)	28 (2·8)	16 (3·0)	136 (5·0)	79 (7·64)	58 (3·54)	45 (9·5)	24 (9·6)
25-29	3396 (19·0)	1,006 (25·1)	173 (17·2)	232 (25·0)	91 (17·6)	45 (10·4)	199 (12·2)	57 (16·5)	75 (10·9)	87 (13·0)	113 (11·4)	101 (18·8)	470 (17·4)	204 (19·73)	358 (21·84)	133 (27·9)	52 (20·8)
30-34	4937(27·6)	1,320 (32·9)	229 (22·8)	264 (28·4)	115 (22·3)	112 (25·8)	357 (21·9)	67 (19·4)	141 (20·6)	176 (26·2)	257 (25·8)	167 (31·2)	731 (27·1)	238 (23·0)	564 (34·41)	134 (28·2)	65 (26·0)
35-39	4041(22·6)	811 (20·2)	224 (22·3)	217 (23·3)	117 (22·7)	110 (25·4)	369 (22·6)	69 (19·9)	144 (21·0)	166 (24·7)	281 (28·2)	140 (26·1)	624 (23·1)	221 (21·4)	393 (24·0)	99 (20·8)	56 (22·4)
40-65	4189 (23·5)	457 (11·4)	287 (28·6)	75 (8·1)	152 (29·5)	144 (33·2)	619 (38·0)	124 (35·8)	275 (40·1)	212 (31·6)	316 (31·8)	112 (20·9)	740 (27·4)	292 (28·24)	266 (16·2)	65 (13·7)	53 (21·2)
Race/Ethnicity †																	
White	11402	3695	182	659	99	113	809	56	476	546	963	519	2585	18	21	446	215
Latina	3695	317	900	167	436	347	982	283	3	179	34	0	25	14	3	4	1

Asian	2187	69	8	9	1	0	3	1	30	2	4	6	19	881	1142	7	5
South Asian	689	19	4	2	1	0	3	0	11	1	0	0	12	170	457	5	4
Black	386	101	6	101	9	0	14	5	114	3	2	0	14	5	10	2	0
Middle Eastern	115	31	6	3	1	0	7	3	5	2	1	5	14	5	25	6	1
Native Hawaiian/ Pacific Islander	47	16	3	2	1	0	3	1	0	3	1	0	1	3	1	4	8
American Indian/ Alaska Native	144	95	5	4	1	2	9	0	0	1	1	0	3	6	17	0	0
Other/Multiracial	558	107	12	61	10	14	36	25	67	13	8	16	70	17	54	21	27
SES (%)																	
Lower-Class	1993 (11·2)	395 (9·8)	106 (10·6)	168 (18·1)	62 (12·0)	45 (10·4)	178 (11·0)	59 (17·1)	68 (9·9)	61 (9·1)	71 (7·1)	55 (10·3)	343 (12·7)	276 (26·7)	58 (3·54)	32 (6·7)	16 (6·5)
Middle-Class	15621 (87·5)	3551 (88·5)	896 (89·2)	756 (81·5)	453 (87·8)	381 (87·8)	1447 (89·0)	285 (82·4)	601 (87·6)	607 (90·6)	919 (92·5)	469 (87·5)	2324 (86·0)	752 (72·7)	1518 (92·62)	435 (91·6)	227 (91·5)
Upper-Class	237 (1·3)	68 (1·7)	2 (0·2)	4 (0·4)	1 (0·2)	8 (1·8)	1 (0·1)	2 (0·6)	17 (2·5)	2 (0·3)	4 (0·4)	12 (2·2)	34 (1·3)	6 (0·6)	63 (3·84)	8 (1·7)	5 (2·0)
Education (%)																	
Never attended school	11 (0·1)	1 (0·02)	1 (0·1)	0	0	0	3 (0·2)	0	2 (0·3)	0	1 (0·1)	0	2 (0·1)	1 (0·1)	0	0	0
Elementary school	141 (0·8)	2 (0·1)	6 (0·6)	13 (1·4)	7 (1·4)	12 (2·8)	52 (3·2)	3 (0·9)	2 (0·3)	26 (3·9)	1 (0·1)	1 (0·2)	11 (0·4)	1 (0·1)	2 (0·1)	2 (0·4)	0
Some high school	946 (5·3)	31 (0·8)	51 (5·1)	36 (3·9)	12 (2·3)	64 (14·8)	104 (6·4)	91 (26·3)	31 (4·5)	81 (12·1)	75 (7·6)	239 (44·6)	60 (2·2)	14 (1·4)	14 (0·9)	21 (4·4)	22 (8·8)
High school graduate or equivalent	2113 (11·9)	308 (7·7)	172 (17·1)	155 (16·7)	97 (18·8)	24 (5·5)	427 (26·3)	10 (2·9)	129 (18·8)	78 (11·6)	240 (24·2)	16 (3·0)	200 (7·44)	104 (10·1)	64 (3·9)	63 (13·3)	26 (10·4)
Some college/university	3160 (17·7)	887 (22·2)	206 (20·5)	182 (19·6)	108 (21·0)	64 (14·8)	350 (21·5)	87 (25·1)	74 (10·8)	47 (7·0)	160 (16·1)	31 (5·8)	480 (17·9)	276 (26·7)	96 (5·9)	73 (15·4)	39 (15·7)
College diploma/university degree	6746 (37·9)	1719 (43·0)	375 (37·4)	261 (28·2)	173 (33·6)	176 (40·7)	433 (26·7)	94 (27·2)	324 (47·2)	224 (33·4)	353 (35·5)	154 (28·7)	1273 (47·34)	489 (47·3)	363 (22·2)	220 (46·3)	115 (46·2)
Professional degree	1690 (9·5)	109 (2·7)	33 (3·3)	210 (22·7)	42 (8·2)	54 (12·5)	202 (12·4)	11 (3·2)	39 (5·7)	157 (23·4)	46 (4·6)	29 (5·4)	272 (10·12)	49 (4·7)	402 (24·6)	16 (3·4)	19 (7·6)
Masters degree	2452 (13·8)	748 (18·7)	140 (13·9)	52 (5·6)	72 (14·0)	33 (7·6)	33 (2·0)	45 (13·0)	69 (10·1)	36 (5·4)	85 (8·6)	52 (9·7)	318 (11·8)	77 (7·5)	601 (36·7)	71 (15·0)	20 (8·0)
Doctoral degree	561 (3·2)	196 (4·9)	20 (2·0)	18 (1·9)	4 (0·8)	6 (1·4)	21 (1·3)	5 (1·5)	16 (2·3)	22 (3·3)	32 (3·2)	14 (2·6)	73 (2·7)	22 (2·1)	95 (5·8)	9 (1·9)	8 (3·2)
Marriage Status (%)																	
Divorced	514 (2·9)	121 (3·0)	36 (3·6)	11 (1·2)	6 (1·2)	18 (4·2)	55 (3·4)	10 (2·9)	41 (6·0)	31 (4·6)	22 (2·2)	23 (4·3)	103 (3·8)	3 (0·3)	25 (1·5)	7 (1·5)	2 (0·8)
Living with a partner	3195 (18·0)	99 (2·5)	210 (21·0)	321 (34·7)	158 (30·7)	129 (29·9)	581 (35·8)	106 (30·6)	55 (8·0)	180 (26·9)	300 (30·2)	29 (5·4)	558 (20·8)	268 (26·0)	14 (0·9)	118 (24·8)	69 (27·8)
Married	12004 (67·4)	3410 (85·3)	596 (59·5)	533 (57·6)	243 (47·3)	207 (47·9)	692 (42·7)	170 (49·1)	474 (69·1)	409 (61·0)	607 (61·2)	463 (86·7)	1597 (59·4)	603 (58·5)	1534 (93·7)	306 (64·4)	160 (64·5)
Never married	1541 (8·7)	332 (8·3)	106 (10·6)	54 (5·8)	72 (14·0)	66 (15·3)	197 (12·2)	33 (9·5)	97 (14·1)	31 (4·6)	32 (3·2)	11 (2·1)	354 (13·2)	96 (9·3)	22 (1·3)	27 (5·7)	11 (4·4)
Separated	437 (2·5)	28 (0·7)	42 (4·2)	6 (0·7)	33 (6·4)	10 (2·3)	73 (4·5)	23 (6·7)	14 (2·0)	18 (2·7)	27 (2·7)	6 (1·1)	59 (2·2)	47 (4·6)	30 (1·8)	15 (3·2)	6 (2·4)

Widowed	110 (0·6)	9 (0·2)	12 (1·2)	1 (0·1)	2 (0·4)	2 (0·5)	24 (1·5)	4 (1·2)	5 (0·7)	1 (0·2)	4 (0·4)	2 (0·4)	16 (0·6)	14 (1·4)	12 (0·7)	2 (0·4)	0

**For variables where categories or observations do not add up to 17871, they were the result of missing responses from participants for the corresponding questions on the survey*

† Race/ethnicity adds up to more than 17871 because respondents were allowed to identify with more than one race/ethnic group, which is why the percentages were not reported

Table 2S: “Big 7” predictors of vaccine acceptance for 16 countries among global pregnant women and mothers of young children

**Association between Exposure Variables and Vaccine Acceptance among Non-Pregnant Women (Likely vs. Unlikely)**	**United States (N=2004; 53% Likely)**		**Argentina (N=1422; 79% Likely)**		**Brazil (N=298; 92% Likely)**		**Chile (N=360; 79% Likely)**		**Colombia (N=467, 86% Likely)**		**Mexico (N=837; 92% Likely)**		**Peru (N=312, 80% Likely)**		**South Africa (N=617, 67% Likely)**	
**Characteristics**	**OR**	**95% CI**	**OR**	**95% CI**	**OR**	**95% CI**	**OR**	**95% CI**	**OR**	**95% CI**	**OR**	**95% CI**	**OR**	**95% CI**	**OR**	**95% CI**
**Past Acceptance and Perceived Safety/Efficacy of Other Vaccines**																
Routine immunization for children (ref: no)	**32·3**	22·1, 47·1	**52·1**	6·8, 400	*	*	**24·3**	5·3, 112	**7·60**	2·82, 20·5	**12·9**	5·74, 29·2	**7·83**	3·08, 19·9	**14·8**	7·90, 27·6
Think childhood vaccines are safe (ref: not safe)	**97·5**	53·1, 179	**29·4**	12·4, 70·2	*	*	**22·2**	7·23, 68·0	**36·4**	11·8, 112	**28·3**	10·3, 77·2	**19·3**	5·26, 70·8	**38·6**	16·4, 90·8
Thinks childhood vaccines are effective in protecting against diseases (ref: not effective)	**168**	62·4, 453	**16·3**	7·70, 34·3	*	*	**83·2**	10·9, 638	**35.0**	9·72, 126	**31.0**	11·5, 83·8	**58·3**	7·42, 459	**27·8**	12·5, 62·0
Did receive flu vaccination last year (ref: did not receive)	**13·5**	10·9, 16·8	**3·58**	2·75, 4·68	**8·66**	3·32, 22·6	**8·35**	4·36, 16·0	**1·79**	1·04, 3·08	**3·39**	2·07, 5·56	**3·57**	2·01, 6·32	**8·59**	5·48, 13·5
**Confidence in COVID-19 Vaccine**																
Confident in safety of COVID-19 vaccine post-approval by country's health agencies (not confident)	**56·6**	39·4, 81·3	**20·1**	12·9, 31·2	**22·5**	7·31, 69·0	**15·3**	7·05, 33·0	**21.0**	9·70, 45·4	**28**	12·6, 62·1	**41·7**	14·6, 119	**117**	53·1, 258
Confident in COVID-19 vaccine efficacy post approval by country's health agencies (not confident)	**29·9**	23·2, 38·6	**22·8**	14·9, 35·0	**33·7**	10·8, 105	**15·6**	7·46, 32·8	**26·5**	12·2, 57·6	**33·9**	15·2, 75·3	**16·2**	7·93, 33·1	**70·1**	39·6, 124
**Perceived Risk of the Virus/Precautions**																
Worried about COVID-19 (not worried)	**19·3**	15·4, 24·2	**6·36**	4·77, 8·47	**32·6**	11·7, 91·1	**16.0**	8·40, 30·4	**8·67**	4·37, 17·2	**6·14**	3·34, 11·3	**6·72**	2·99, 15·1	**8·8**	6·00, 12·9
Important for country to have vaccine (not important)	**77·2**	50·8, 117	**16·7**	9·81, 28·4	**72·8**	14·2, 373	**5·27**	2·09, 13·3	**10·2**	4·44, 23·34	**3·73**	1·85, 7·50	**11·7**	3·54, 38·9	**49·6**	25·6, 96·0
Important for the majority of people in their country getting vaccinated (not important)	**66.0**	47·8, 91·0	**21.0**	14·0, 31·3	**29·6**	7·86, 112	**18·9**	6·79, 52·8	**48·3**	15·9, 146	**5·83**	3·09, 11·0	**15·9**	4·99, 50·9	**48·5**	26·9, 87·5
Closely complied with mask guidelines (ref: did not comply)	**36·7**	22·0, 61·2	**9·88**	3·08, 31·7	**19·1**	3·98, 91·5	**16.0**	1·76, 145	**3·05**	0·27, 34·08	**3·77**	0·75, 19·0	*	*	*	*
**Monitoring COVID-19 news on any media (ref: never)**																
At least once in a while	**11·3**	7·78, 16·5	**5·86**	4·04, 8·49	**15·5**	5·71, 41·9	**4·80**	2·15, 10·7	**2·42**	1·11, 5·27	**4·64**	2·14, 10·1	**6·10**	1·87, 19·9	**3·57**	2·16, 5·91
**Public Trust and Satisfaction**																
Trust in PH Agencies (ref: no trust in PH agencies)	**16·6**	13·2, 20·9	**7·57**	5·54, 10·4	**14·4**	4·73, 43·8	**7·68**	4·18, 14·1	**9·73**	5·25, 18·06	**8·92**	5·08, 15·6	**6·94**	3·70, 13·0	**19·2**	11·8, 31·2
Trust in science (ref: no trust in science)	**15·5**	11·9, 20·2	**5·49**	4·09, 7·38	**10·5**	4·19, 26·6	**4·54**	2·62, 7·88	**6·02**	3·48, 10·4	**4·26**	2·53, 7·17	**4·13**	2·29, 7·44	**8·48**	5·77, 12·5
Increased trust in science during COVID-19 (ref: no increased trust in science)	**12·8**	9·52, 17·1	**6·82**	5·06, 9·21	**14·8**	4·25, 51·2	**3·82**	2·17, 6·72	**7·33**	4·06, 13·2	**3·55**	2·14, 5·90	**4·58**	2·47, 8·49	**10·03**	6·35, 15·8

Association between Exposure Variables and Vaccine Acceptance among Non-Pregnant Women (Likely vs. Unlikely)	Italy (N=758, 77% Likely)		Russia (N=383, 48% Likely)		Spain (N=484, 76% Likely)		United Kingdom (1911; 64% Likely)		India (N=1386; 93% Likely)		Philippines (N=943; 80% Likely)		Australia (N=220, 55% Likely)		New Zealand (N=160, 66% Likely)	
**Characteristics**	**OR**	**95% CI**	**OR**	**95% CI**	**OR**	**95% CI**	**OR**	**95% CI**	**OR**	**95% CI**	**OR**	**95% CI**	**OR**	**95% CI**	**OR**	**95% CI**
**Past Acceptance and Perceived Safety/Efficacy of Other Vaccines**																
Routine immunization for children (ref: no)	**22·9**	10·9, 48·1	**17·2**	8·34, 35·6	**9·12**	**2·38, 35·0**	**29·8**	17·1, 51·9	**10·5**	6·1, 18·1	**4·24**	2·63, 6·85	*	*	**84.0**	10·9, 647
Think childhood vaccines are safe (ref: not safe)	**22·4**	12·5, 40·2	**13·7**	7·64, 24·6	**59·5**	7·79, 454	**49·9**	21·4, 78·0	**30·8**	16·1, 59·1	**13·9**	6·16, 31·4	*	*	*	*
Thinks childhood vaccines are effective in protecting against diseases (ref: not effective)	**46·8**	18·3, 120	**14·8**	7·38, 29·7	**23·4**	5·19, 105	**71.0**	29·0, 174	**43·2**	20·5, 91·0	**12·3**	6·20, 24·4	*	*	*	*
Did receive flu vaccination last year (ref: did not receive)	**7·84**	4·78, 12·8	**8·76**	4·80, 16·0	**2·24**	1·36, 3·68	**7·12**	5·78, 8·78	**1·96**	1·29, 3·00	**1·84**	1·29, 2·64	**29·1**	14·0, 60·5	**10·2**	4·71, 22·3
**Confidence in COVID-19 Vaccine**																
Confident in safety of COVID-19 vaccine post-approval by country's health agencies (not confident)	**49·1**	24·5, 98·4	**15·3**	5·96, 39·3	**27·4**	9·88, 75·7	**67·7**	45·9, 99·8	**14·6**	8·96, 23·7	**15·8**	10·1, 24·6	**490**	64·5, 3718	**88·8**	20·1, 392
Confident in COVID-19 vaccine efficacy post approval by country's health agencies (not confident)	**32·1**	19·1, 53·8	**15·2**	7·34, 31·6	**26·5**	11·4, 61·9	**35·1**	26·8, 46·1	**16·1**	10·1, 25·7	**13·8**	9·3, 20·5	**450**	123, 1639	**40·7**	15·8, 105
**Perceived Risk of the Virus/Precautions**																
Worried about COVID-19 (not worried)	**10·8**	6·78, 17·2	**8·13**	5·03, 13·2	**12·1**	6·72, 21·6	**9·36**	7·53, 11·6	**6·05**	3·88, 9·45	**5·93**	3·78, 9·33	**19·7**	9·38, 41·2	**5·75**	2·80, 11·8
Important for country to have vaccine (not important)	**70·8**	31·7, 158	**58·4**	22·9, 149	**6·82**	2·02, 23·1	**55·4**	35·4, 86·7	**99·4**	37·0, 267	**32·8**	15·2, 70·9	**413**	54·5, 3123	**43·4**	13·84, 136
Important for the majority of people in their country getting vaccinated (not important)	**78·4**	40·1, 154	**54·3**	25·1, 117	**21·3**	7·94, 56·9	**57·3**	39·4, 83·3	**81·5**	34·4, 193	**41·7**	20·2, 86·0	**161**	46·0, 562	**31·9**	12·5, 81·1
Closely complied with mask guidelines (ref: did not comply)	*	*	**32·0**	11·4, 89·9	*	*	**32.0**	16·7, 61·3	**1·71**	0·39, 7·56	**5·44**	1·87, 15·9	**21·5**	4·51, 102	**11·3**	3·33, 38·3
**Monitoring COVID-19 news on any media (ref: never)**																
At least once in a while	**28·2**	6·38, 125	**2·86**	1·81, 4·53	**6·18**	2·03, 18·8	**6·31**	4·32, 9·24	**3·5**	1·91, 6·41	**2·23**	0·74, 6·73	**10·1**	3·37, 30·4	**13·3**	2·83, 62·5
**Public Trust and Satisfaction**																
Trust in PH Agencies (ref: no trust in PH agencies)	**12·7**	8·47, 19·0	**13·6**	5·68, 32·5	**5·37**	3·34, 8·63	**19·88**	15·5, 25·4	**8·24**	5·22, 13·01	**5·56**	3·92, 7·88	**161**	57·9, 450	**30·8**	11·8, 80·2
Trust in science (ref: no trust in science)	**9·28**	6·17, 14·0	**4·19**	2·66, 6·61	**4·18**	2·62, 6·66	**8·96**	7·13, 11·2	**5·25**	3·31, 8·32	**5·10**	3·57, 7·28	**61·6**	18·3, 209	**12·4**	3·90, 39·3
Increased trust in science during COVID-19 (ref: no increased trust in science)	**7·65**	5·09, 11·5	**6·41**	3·54, 11·6	**5·47**	3·31, 9·06	**8·15**	6·14, 10·8	**5·28**	3·42, 8·13	**4·23**	3·03, 5·90	**21·6**	8·77, 53·2	**6·15**	2·91, 13·0

**Dropped: observations were dropped when all the participants in one category were unlikely to accept COVID-19 vaccine*
